# Intimate partner violence during COVID-19: systematic review and meta-analysis according to methodological choices

**DOI:** 10.1186/s12889-024-17802-9

**Published:** 2024-01-29

**Authors:** Diogo Costa, Florian Scharpf, Alexa Weiss, Arin H. Ayanian, Kayvan Bozorgmehr

**Affiliations:** 1 Department of Population Medicine and Health Services Research, School of Public Health, Bielefeld University, P.O. Box: 10 01 31, 33501 Bielefeld, Germany; 2 Institute for Interdisciplinary Research On Conflict and Violence (IKG), Bielefeld University, Bielefeld, Germany; 3 Department of Psychology, Bielefeld University, Bielefeld, Germany; 4 Department of General Practice and Health Services Research, Section for Health Equity Studies and Migration, Heidelberg University Hospital, Heidelberg, Germany; 5 Research Centre for Human Development (CEDH), Faculty of Education and Psychology, Universidade Católica Portuguesa, Porto, Portugal; 6 Yerevan State University, Yerevan, Armenia

**Keywords:** Intimate partner violence, COVID-19, Methods, Prevalence, Systematic review

## Abstract

**Background:**

Intimate Partner Violence (IPV) is the most common form of interpersonal violence and a major public health problem. The COVID-19 pandemic might have contributed to an increase in IPV experiences. To evaluate changes in IPV prevalence during the pandemic, it is important to consider studies’ methodological characteristics such as the assessment tools used, samples addressed, or administration modes (e.g., face-to-face, telephone or online interviews), since they may influence disclosure and were likely affected by pandemic-imposed mobility restrictions.

**Methods:**

Systematic review and meta-analysis of empirical studies addressing IPV against women, men, or both, during the COVID-19 period. We searched six electronic databases until December 2021, including articles in English, German, Spanish, French or Portuguese languages. We extracted and synthesised characteristics of studies related to sampling (clinical, community, convenience), type assessment tool (standardised questionnaire, specifically created questions), method of administration (online, telephone, face-to-face), and estimates of different forms of IPV (physical, sexual, psychological). IPV estimates were pooled stratified by study characteristics using random-effects models.

**Results:**

Of 3581 publications, we included 103 studies. Fifty-five studies used a standardized instrument (or some adaptations) to assess IPV, with the World Health Organisation Questionnaire and the Revised Conflicts Tactics Scales being the most frequent. For 34 studies, the authors created specific questions to assess IPV. Sixty-one studies were conducted online, 16 contacted participants face-to-face and 11 by telephone. The pooled prevalence estimate for any type of violence against women (VAW) was 21% (95% Confidence Interval, 95%CI = 18%-23%). The pooled estimate observed for studies assessing VAW using the telephone was 19% (95%CI = 10%-28%). For online studies it was 16% (95%CI = 13%-19%), and for face-to-face studies, it was 38% (95%CI = 28%-49%). According to the type of sample, a pooled estimate of 17% (95%CI = 9%-25%) was observed for studies on VAW using a clinical sample. This value was 21% (95%CI = 18%-24%) and 22% (95%CI = 16%-28%) for studies assessing VAW using a convenience sample and a general population or community sample, respectively. According to the type of instrument, studies on VAW using a standardized tool revealed a pooled estimate of 21% (95%CI = 18%-25%), and an estimate of 17% (95%CI = 13%-21%) was found for studies using specifically created questions.

**Conclusions:**

During the pandemic, IPV prevalence studies showed great methodological variation. Most studies were conducted online, reflecting adaptation to pandemic measures implemented worldwide. Prevalence estimates were higher in face-to-face studies and in studies using a standardized tool. However, estimates of the different forms of IPV during the pandemic do not suggest a marked change in prevalence compared to pre-pandemic global prevalence estimates, suggesting that one in five women experienced IPV during this period.

**Supplementary Information:**

The online version contains supplementary material available at 10.1186/s12889-024-17802-9.

## Background

As a result of the COVID-19 pandemic, several control measures, including lockdowns and stay-at-home regulations with different stringency levels were imposed worldwide. These may have contributed to an increase in the rates of intimate partner violence (IPV)—the most frequently experienced form of interpersonal violence worldwide, particularly among women [1] – by reducing the chances of victims escaping or accessing help, by increasing exposure to abusive partners, and by exacerbating risk factors for perpetration (e.g. alcohol consumption). Intimate partner violence refers to behaviour within an intimate relationship, including acts of physical aggression, sexual coercion, psychological abuse and controlling behaviours, happening between current or former spouses and partners, that cause physical, sexual or psychological harm, [2].

Several reports and researchers suggest that IPV has increased during lockdown periods [3, 4], although reported cases to authorities showed mixed results [5–7]. This variation can reflect true geographical and cultural nuances, different effects of interventions taken to counter IPV, or methodological differences known to influence IPV reporting.

Observed IPV rates vary widely according to the mode of enquiry (standardized questionnaire, single item, interview) [8], the administration method (face-to-face, self-administered, computer-based) [9] or the definitions used [8]. Furthermore, the data sources used or the type of samples assessed (e.g., general population-based samples used in surveys, or crime victimization studies drawing from judicial or police records) strongly influence the variation found in the frequency of different types of domestic violence (sexual, psychological, physical) and the gender (a)symmetry of victimization and perpetration rates [10]. Importantly, the methodological specificities of studies, including the sampling sources, may be systematically impacted by the mobility restrictions imposed during the pandemic, and therefore need to be considered when trying to gauge changes in IPV frequency during the pandemic. In particular, mobility restrictions plausibly hindered participation in face-to-face surveys, hampered the ability of victims to complain (which could be reflected in rates obtained through official administrative victim records, such as police records), or promoted reporting through helplines, provided victims could still reach such lines during the strict lockdowns observed. A 2021 review conducted in 11 Western and Southern European countries showed considerable variation in IPV frequency changes during the COVID-19 pandemic, with six countries showing increases, two countries showing decreases, two without changes and one where changes were unclear [11]. Importantly, within the same country, there sometimes were increases in helpline calls for IPV but simultaneous decreases in police reports (e.g. Spain). In contrast, in other countries there were simultaneous decreases in both police reports and helplines (e.g. Italy). Thus, to shed light on the potential impact of (measures taken to contain) the COVID-19 pandemic on IPV prevalence estimates during the SARS-Cov-2 pandemic in a comprehensive review, empirical studies must be qualified according to their methodological characteristics (acknowledging differences, e.g., in data collection methods and different reporting systems) before meaningful comparisons can be established.

This systematic review aims to synthesize the evidence about IPV frequency estimates measured during the SARS-Cov-2 pandemic and detail the main methodological characteristics of such studies to provide a clearer picture of the variation in IPV frequency, by groups of methodologically comparable studies and samples. Findings from this systematic review could have implications for future decisions on public health measures in the context of pandemics and inform researchers examining the epidemiology of IPV.

## Methods

### Search strategy and selection criteria

The following research questions guided this systematic review and meta-analysis:

- What was the prevalence of intimate partner violence victimization during the COVID-19 pandemic among adults?
- What were the main methodological characteristics of the studies assessing IPV during the COVID-19 pandemic (e.g. administration methods, instruments used, sampling frameworks, etc.)?
- How do prevalence or incidence IPV estimates during the COVID-19 pandemic vary according to the study’s main methodological characteristics?

The protocol for this review was registered in PROSPERO database (CRD42021297362) and followed recommendations for systematic reviews in health promotion and public health [12].

To answer the review questions, we conducted a systematic search of scientific databases using MEDLINE via PubMed and Web of Science Core Collection; WHO COVID-19 database; the Cochrane Library; PsychInfo and CINAHL via EBSCO. We developed the search strategy according to the Cochrane Handbook for Systematic Reviews of Interventions [13]. The search was performed in the title, abstract and keywords of articles. Several search terms were combined using different Boolean operators, truncations, phrases, and filters. The search comprised a combination of free text search terms and subject headings (MeSH) related to the following main concepts: 1) coronavirus disease 2019 (COVID-19) caused by an infection with SARS-CoV-2; and 2) domestic violence, intimate partner violence, partner abuse. The search strategy details are presented as supplementary material (Table S1).

A PICO framework was used to help define the inclusion/exclusion criteria: population was defined as adult (18 years old or above) men and women from studies conducted among samples drawn from the general population, community samples or drawn from any selected/convenience or clinical or sheltered sample. As eligible study designs we considered all empirical quantitative, qualitative and mixed-methods studies documenting intimate partner violence during the COVID-19 pandemic, including, but not limited to, studies about interventions enacted towards intimate partner violence. The main outcome of interest was exposure to any form of intimate partner violence during the COVID-19 pandemic (including the forms defined by the WHO, i.e., any act of psychological, sexual, and physical violence, as well as any other forms described [1]) and measured as prevalence or incidence. Eligible studies could also refer to domestic violence (DV), partner abuse, battered women/men, or any other definition fitting the criteria for this type of interpersonal violence, as long as the victim-perpetrator relationship (i.e., a current or previous intimate relationship) was possible to identify.

Finally, we included studies in English, German, Spanish, French or Portuguese, published in peer-reviewed journals, indexed from December 1st, 2019 (date of the first Covid-19 outbreak in Wuhan, China), onwards were included. Searches were performed on December 9th 2021.

### Screening

Four independent researchers screened the titles and abstracts applying the pre-defined inclusion/exclusion criteria, using the web-based tool Covidence (https://www.covidence.org/). Full texts were obtained for all included studies and for those where no agreement could be established based on title and abstract.

**Fig. 1:**
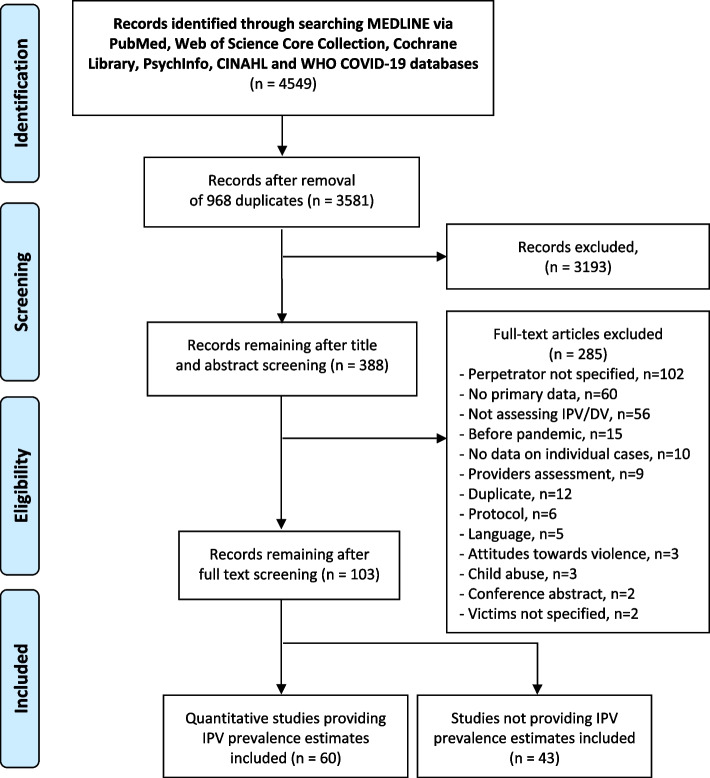
PRISMA flow diagram

Relevant parameters were extracted, including any prevalence or incidence IPV estimate measured among adult women, men, or both, and according to violence types: physical, psychological (i.e., verbal, emotional), sexual, or “any” if not described. Methodological characteristics of studies extracted included type of sample (general population/community sample), sample size, tool or instrument used for IPV assessment, method of administration (face-to-face interviews, telephone, online, other), and other relevant study characteristics (PRISMA Flowchart presented in Fig. 1). We also retrieved any narrative description of a change (increase/decrease) in IPV during the pandemic or any reported difference in estimates between the pre and post pandemic periods reported (presented as Supplementary material, Table S3).

The quality of studies was assessed using Joanna Briggs Institute critical appraisal tools [14], with the appropriate checklists according to the study designs included (conducted by the first author only). The quality scores were calculated as a total out of the maximum number of applicable questions and computed as a percentage. Studies with a score of 75–100% were considered high quality, 50–74% of moderate quality and 0–49% low quality (final scores presented in Supplementary material, Table S4). This resulted in 67 studies appraised with high quality, 25 as moderate, and 11 as low.

### Data analysis

We extracted relevant parameters from quantitative studies to synthesise a pooled measure of IPV prevalence. We stratified the pooling according to:

- intimate partner violence against women (VAW), both women and men (VAW&M) or men only (VAM);
- IPV type, in “any”, physical, psychological, sexual (results for physical, psychological and sexual IPV are presented in Supplementary material, Figures S1 to S9);
- the main methodological choices, precisely the method of administration (face-to-face, online, telephone, other), the type of sample (general population/community sample, convenience sample, clinical sample, other), and the instrument (use of a standardized tool, specifically created or purpose-built questions by authors, or other such as clinical records consulted).

For the meta-analysis, we assumed that prevalences are randomly sampled from a distribution and thus applied a random-effects approach. Between-study variance, *τ*^2^, was calculated instead of I^2^ as measure of heterogeneity following published recommendations [15], and displayed in the caption of each forest plot. Risk of bias and publication bias were assessed through funnel plots (presented in Supplementary material, Figure S10). Forests plots restricted to studies conducted in the USA (given the preponderance of studies from this region, *n* = 30) are presented as Supplementary material (Figures S2.1 to S2.9). Forest plots restricted to studies appraised with high quality (*n* = 67) are also presented as Supplementary material (Figures S3.1 to S3.9).

Stata V16 with commands *metaprop* and *meta* were used for the analysis.

## Results

After duplicates removal, we screened 3581 titles and abstracts for eligibility. We included a total of 388 studies for full text-screening and 103 in the analysis.

We extracted and recorded information from all quantitative studies according to the available data, which varied in terms of the outcomes (different types of IPV) and methodological choices described. From studies using a qualitative type of design, we retrieved the relevant methodological characteristics of IPV assessment.

Study characteristics are shown in Table 1. The complete list including tools used can be consulted in the supplementary material (Table S2). Studies were included from all world regions, but studies from the USA were the most common (*n* = 30 or 29%). Furthermore, five studies were from India, and four studies each were from Ethiopia, Iran and Spain.

**Table 1:** Studies characteristics

Methodological Characteristics		**n (%)**
Assessing violence against women (VAW)		51 (49.5)
Assessing violence against men (VAM)		3 (2.9)
Assessing violence against women and men (VAW&M)		49 (47.6)
Sample size (mean, standard deviation)		1934927 (18939980)
Median sample size of studies on VAW		544
Median sample size of studies on VAM		86
Type of sample	General population/community	24 (23.3)
	Convenience	56 (54.4)
	Clinical	18 (17.4)
	Other	5 (4.9)
Assessment method	Standardized instrument	55 (53.4)
	Specific created questions	34 (33.0)
	Other	14 (13.6)
Method of administration	Online questionnaire	61 (59.3)
	Face-to-face interviews	16 (15.5)
	Telephone	11 (10.7)
	Other	15 (14.6)

The median sample size of studies on VAW was 544 and on VAM was 86 participants/records per study. A total of 56 studies used convenience samples (either referring directly as convenient or not detailing any sampling frame or sampling strategy allowing to characterize the sample as drawn through a probabilistic approach), 24 used samples from the general population or community, and 18 resorted to clinical samples.

### Methodological characteristics

Overall, 55 studies referred the use of a standardized instrument (or adaptations from) to assess IPV. The World Health Organisation (WHO) Questionnaire [16] was the most commonly referred (by at least 14 studies included), followed by the Revised Conflicts Tactics Scales [17] (by at least 8 studies). Other instruments used in the studies included were: The CAS-SF—Composite abuse scale (revised)—short form [18]; Domestic Violence Against Women Scale (DVAWS) [19]; Psychological Maltreatment of Women Inventory [20]; The Violence Exposure Scale (VES) [21]; Jellinek inventory for assessing partner violence (J-IPV) [22]; UN-MENAMAIS Study questionnaire [23]; Coping Using Sex Inventory (CUSI) [24]; Women’s Experience with Battering [25]; Abuse Behavior Inventory [26]; Conflicts and Problems-Solving Scales (CPS) [27]; Domestic Violence Questionnaire (DVQ) [28]; Abusive Behavior Inventory-R2 (ABI-R2) [29]; Family Maltreatment Measure [30]; Hurt-Insult-Threaten-Scream (HITS) [31]; Epidemic–Pandemic Impacts Inventory (EPII) [32]; Cyber Aggression in Relationships Scale (CARS) [33]; DOORS family violence screening tool [34]; Personal Safety Survey [35]; HARK questionnaire [36]; Cyber Dating Abuse Questionnaire (CDAQ) [37]; Universal Violence Prevention Screening Protocol [38]; and the Abuse Assessment Screen [39].

For 34 studies, the authors created ad-hoc instruments for the purpose of their study, such as specifically created single items or scales, to assess IPV and 14 resorted to other types of assessment (e.g. consulted clinical records).

A total of 61 studies were conducted online, 16 studies reported face-to-face contact with participants (this could include self-administration pencil and paper questionnaires, presented by a physically present interviewer or researcher), and 15 resorted to another method of administration or did not provide sufficient details (e.g. consulted existent clinical or police records).

Most studies included an assessment of IPV experienced during the pandemic period, but 11 studies referred specifically to experiences during the past year. The specific timing or referent period of IPV assessment is provided in the supplementary material (Table S2), for all included studies specifying this information.

### Prevalence of IPV according to methodological choices

**Fig. 2:**
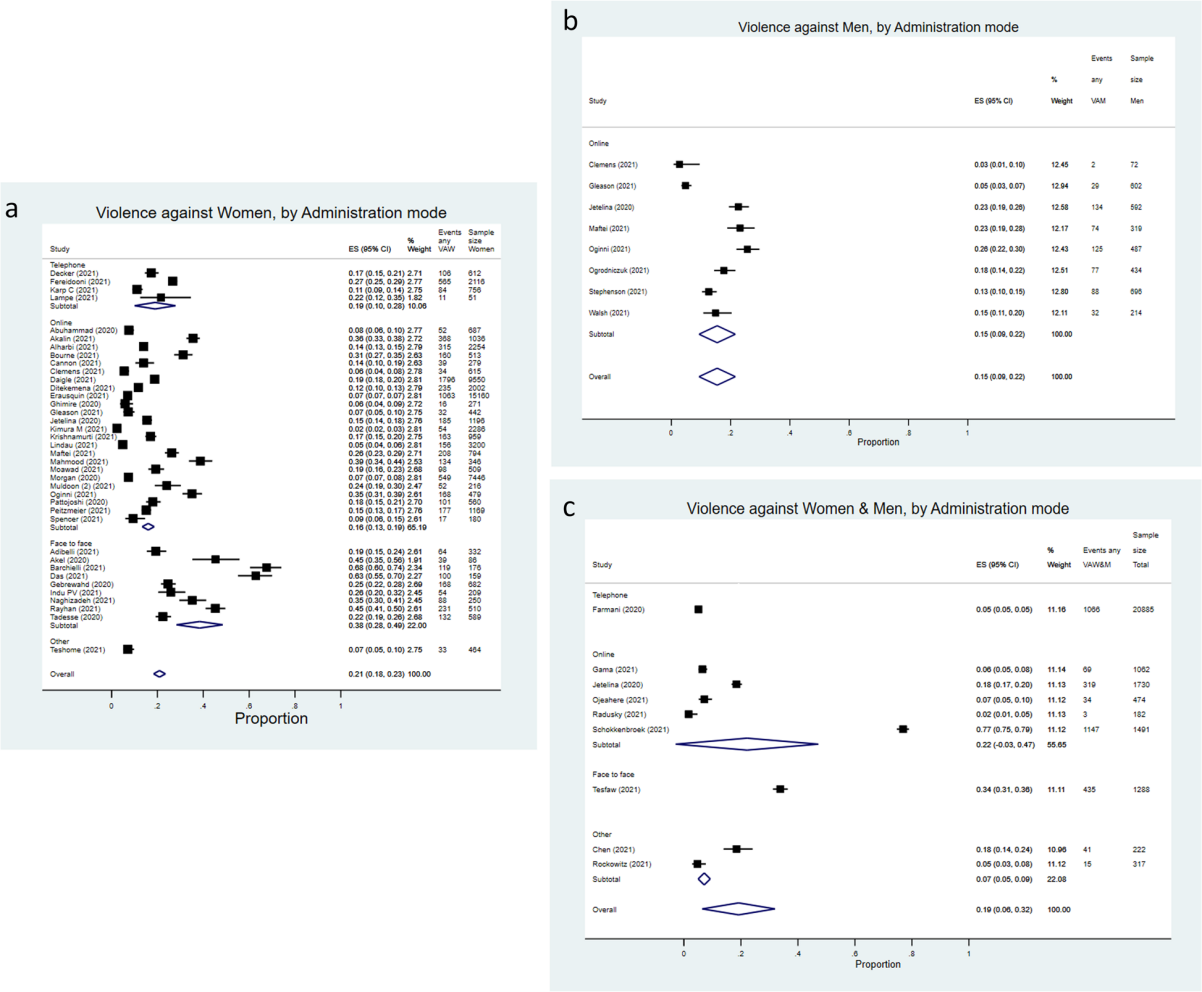
**a** Forest plot of (“any” type of) intimate partner violence against women (VAW) prevalence pooled by method of administration in telephone, online, face-to-face, or other, between-study variance, τ^2^ = 0.00609. I^2^: Telephone = 97.32%, *p* = 0.00; Online = 99.04%, *p* = 0.00; Face to face = 97.33%, *p* = 0.00; Overall = 99.04%, *p* = 0.00. **b** Forest plot of (“any” type of) intimate partner violence against men (VAM) prevalence pooled by method of administration (all were conducted “online”), between-study variance, τ^2^ = 0.00748. I^2^: Online = 96.67%, *p* = 0.00. **c** Forest plot of (“any” type of) intimate partner violence against women and men prevalence pooled by method of administration in telephone, online, face-to-face, or other (from studies where sex-disaggregated prevalence estimates were not available, VAW&M), between-study variance, τ^2^ = 0.03787. I^2^: Online = 99.89%, *p* = 0.00; Overall = 99.84%, *p* = 0.00

For studies assessing violence against women (VAW), the overall pooled prevalence estimate for any type of violence reported was 21% (95% Confidence Interval, 95%CI = 18%-23%), as shown in Fig. 2a. According to method of administration, the pooled estimate observed for studies assessing VAW using the telephone as method of administration was 19% (95%CI = 10%-28%), for studies conducted online the pooled estimate was 16% (95%CI = 13%-19%), and for studies approaching participants face-to-face the pooled estimate was 38% (95%CI = 28%-49%).

In studies assessing violence against men (VAM), estimates were available only from studies conducted online (Fig. 2b), with a pooled prevalence estimate of 15% (95%CI = 9%-22%).

For studies assessing violence against women and men (or not disaggregating estimates by sex, Fig. 2c), the pooled prevalence estimate obtained was 19% (95%CI = 6%-32%), with great variation for those conducted online (pooled estimate = 22%, 95%CI = 3%-47%, with one of the studies providing an estimate of 77% for any type of violence).

**Fig. 3:**
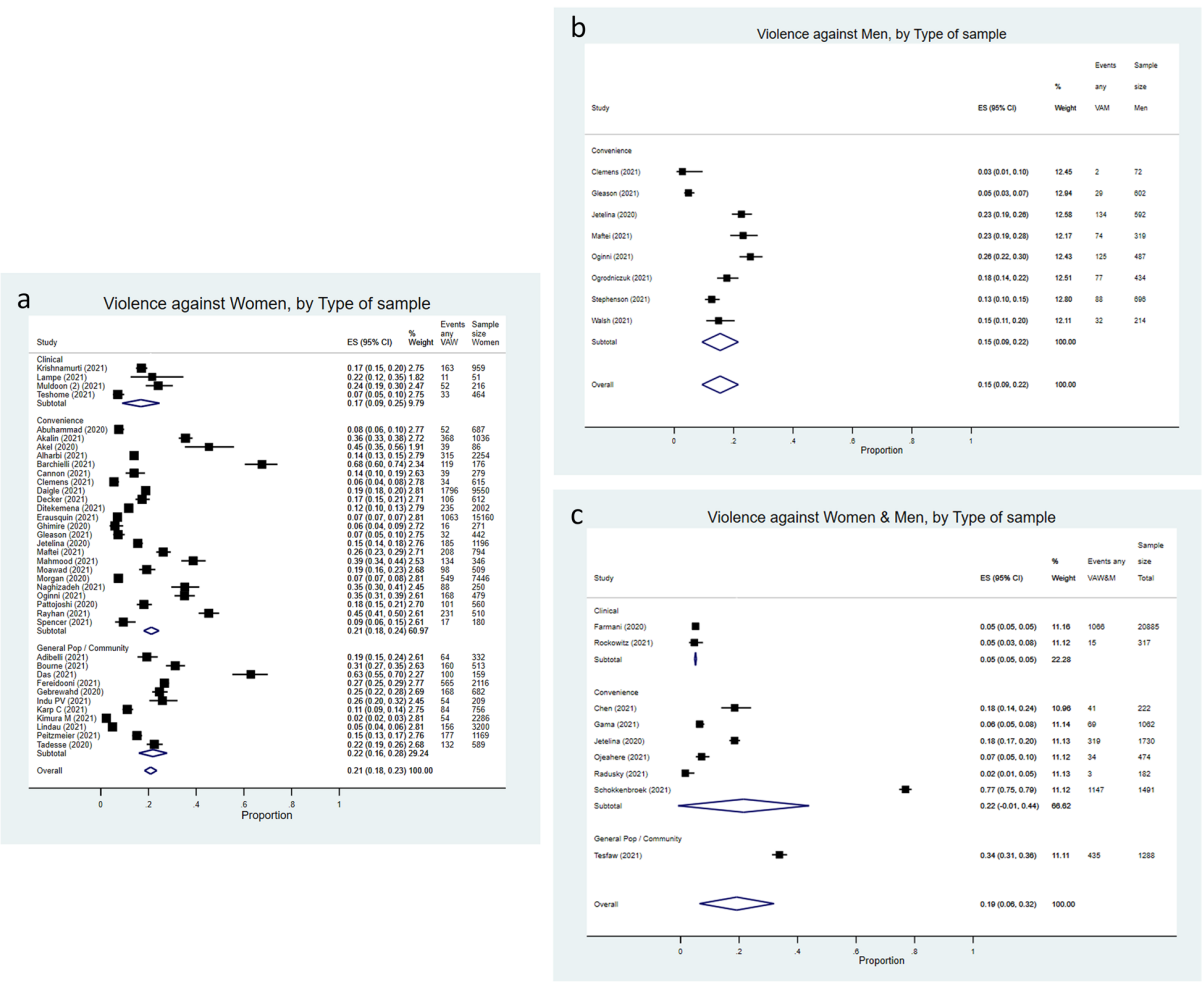
**a** Forest plot of (“any” type of) intimate partner violence against women (VAW) prevalence pooled by type of sample in clinical, convenience or general population/community sample, between-study variance, τ^2^ = 0.00609. I^2^: Clinical = 94.22%, *p* = 0.00; Convenience = 99.02%, *p* = 0.00; General pop/community = 99.26%, *p* = 0.00; Overall = 99.04%, *p* = 0.00. **b** Forest plot of (“any” type of) intimate partner violence against men (VAM) prevalence pooled by type of sample (all used a “convenience” sample), between-study variance, τ^2^ = 0.00748. I^2^: Convenience = 96.67%, *p* = 0.00. **c** Forest plot of (“any” type of) intimate partner violence against women and men prevalence pooled by type of sample in clinical, convenience or general population/community sample (from studies where sex-disaggregated prevalence estimates were not available, VAW&M), between-study variance, τ^2^ = 0.03787. I^2^: Convenience = 99.86%, *p* = 0.00; Overall = 99.84%, *p* = 0.00

According to the type of sample, a pooled estimate of 17% (95%CI = 9%-25%) was observed for studies on VAW using a clinical sample (Fig. 3a). This value was 21% (95%CI = 18%-24%) and 22% (95%CI = 16%-28%) for studies assessing VAW using a convenience sample and a general population or community sample, respectively. For studies focusing on VAM, we observed the same result as the one obtained for the stratification by method of administration, since the same studies were included (Fig. 3b).

For studies focusing on violence against both women and men (Fig. 3c), the two studies conducted on clinical samples resulted in a pooled estimate of 5%, while the studies using a convenience sample showed the same pooled estimate of 22% (same studies as the ones employing an online study as a method of administration, see above).

**Fig. 4:**
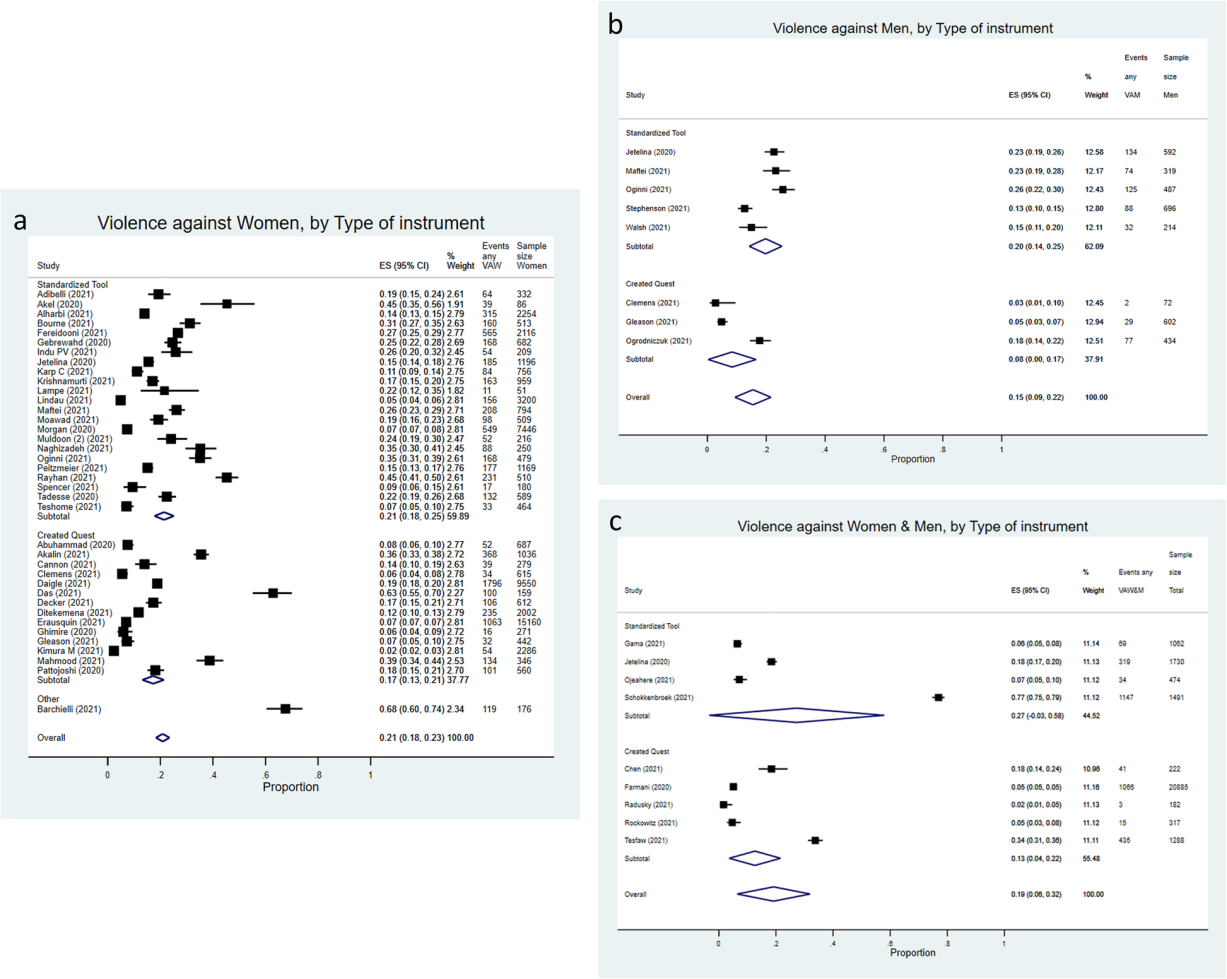
**a** Forest plot of (“any” type of) intimate partner violence against women prevalence pooled by instrument used for assessment in standardized tool, specifically created questions, or other, between-study variance, τ2 = 0.00609. I^2^: Standardized tool = 98.63%, *p* = 0.00; Created Quest = 99.30%, *p* = 0.00; Overall = 99.04%, *p* = 0.00. **b** Forest plot of (“any” type of) intimate partner violence against men prevalence pooled by instrument used for assessment in standardized tool and specifically created questions or other, between-study variance, τ^2^ = 0.00748. I^2^: Standardized tool = 91.41%, *p* = 0.00; Overall = 96.67%, *p* = 0.00. **c** Forest plot of (“any” type of) intimate partner violence against women and men prevalence pooled by instrument used for assessment in standardized tool and specifically created questions or other (from studies where sex-disaggregated prevalence estimates were not available), between-study variance, τ^2^ = 0.03788. I^2^: Standardized tool = 99.90%, *p* = 0.00; Created Quest = 99.21%, *p* = 0.00; Overall = 99.84%, *p* = 0.00

According to the type of instrument (Fig. 4a), studies on VAW using a standardized tool revealed a pooled estimate of 21% (95%CI = 18%-25%). When specifically created questions were used, an estimate of 17% emerged (95%CI = 13%-21%). Only one study used “other” type of assessment instrument and provided an IPV estimate (68%).

For studies assessing VAM and resorting to a standardized tool for IPV assessment, a pooled estimate of 20% (95%CI = 14%-25%) was noted (Fig. 4b). For those assessing VAM and using specifically created questions, the estimate was 8% (95%CI = 0–17%).

Finally, studies focusing on both women and men (VAW&M) and using a standardized tool, a pooled estimate of 27% (95%CI = -3%-58%) was observed (Fig. 4c). Specifically created questions resulted in a pooled estimate of 13% (95%CI = 4%-22%), among this group of studies.

Forest plots for studies providing estimates according to IPV types (physical, psychological, and sexual), are presented as Supplementary material (Figures S1 to S9), only for studies focusing on violence against women (studies focusing on men or both sexes did not provide enough estimates to compute pooled prevalences). Funnel plots for all analyses are presented also as Supplementary material (Figure S10).

## Discussion

This systematic review and meta-analysis shows that publications assessing IPV during the Covid-19 pandemic exhibit great methodological variation. The most frequent methodological features suggested a tendency to use convenient types of samples (compared to probabilistic random samples), assessing IPV through standardised questionnaires (notably the WHO Questionnaire and the Revised Conflicts Tactics Scales), and using online administration.

The online feature appears to be a direct result of lockdowns imposed to mitigate the spread of SARS-Cov-2 infections and might have influenced the results of studies assessing IPV, which rely on self-disclosure of sensitive experiences. Of the 61 studies identified in this review as conducted online, 75% were classified as using a convenience sample. This suggests a methodological tendency observed during the pandemic. The lockdown experience increased the time that potential victims and perpetrators spent together, thus increasing the opportunities for violence episodes. Online surveys might have captured such phenomenon provided that victims were able to disclose such episodes privately through available devices with internet access. However, the privacy needed for a victim to fill in a sensitive questionnaire about her/his experiences of violence, might not have been sufficient or ideal during lockdown. While the overall pooled prevalence found in this study was in line with previous global estimates of violence [2, 40], (pointing to 21% for VAW), the pooled prevalence for studies assessing VAW online was slightly lower (16%), which may suggest an impact on disclosure particularly among women. Face-to-face studies showed higher prevalence estimates in studies of VAW, which may reflect the requirements often found in studies assessing IPV using interviewers i.e. thorough interviewer training, privacy settings, aiming to maximize disclosure while ensuring safety and support if needed [41]. This is reinforced by the results obtained when stratifying according to types of violence: studies on VAW assessing physical violence show the same trend, with face-to-face mode presenting a higher pooled prevalence (16%) compared to the other administration modes (Supplementary Figure S1).

A study conducted in Germany that assessed three representative population surveys, through face-to-face interviews, comparing prevalence before and after the implementation of the “infection control” measures in the country, found no statistically significant increase in physical or sexual IPV as a result of the implementation of control measures [42]. Their results suggest a (12 month) prevalence of physical IPV victimization of 6.4% for female and of 7.0% for male participants and for sexual violence of 3.2% for female and 0.1% for male, in 2021. This is a lower prevalence for population-based studies compared to our pooled estimate for this type of sample and types of violence (only studies in VAW included: physical 10%, Figure S4 supplementary material, and sexual: 9%, Figure S6 supplementary material), and for this type of administration mode (physical VAW: 16%—Figure S1 in supplementary material, and sexual VAW: 12%, Figure S3 supplementary material).

In our results, the sample type (clinical, convenience, or community) did not reveal major differences in pooled estimates on studies focusing on VAW, VAM or VAW&M, but only a slightly lower prevalence for clinical types of samples. In the case of convenient samples, we would expect higher prevalence estimates due to the less controlled sampling procedure used, which could lead to greater self-selection among these types of samples. However, this was not observed. In the case of clinical samples, the results may reflect the tendency to use shorter instruments when assessing IPV among clinical samples, or instruments that focus only on one form of violence (e.g. physical, or sexual), since studies conducted in such settings are often designed to assess clinical features in relation to several associated factors and not IPV exclusively. We expected higher prevalence among clinical samples, which included patients attended in different specialties including emergency departments, and pregnant women. The fact that we did not find marked differences in IPV frequencies among clinical samples compared to other types of samples, suggests also the need to include routine screening for violence during clinical encounters, since these encounters may be the victim’s first opportunity to disclose their experiences, and victims often refrain from actively disclosing due to different reasons including shame and, fear of retaliation [43].

When considering the type of instrument used to assess IPV, standardized tools (such as the WHO tool, or the Revised Conflict Tactics Scales—CTS2) showed higher prevalences than specifically created questions (for both VAW and VAM studies). This may be the result of the behaviour-based type of assessment, which asks respondents to report the frequency of concrete acts of violence. The latter might not always be covered by specifically created questions, tailored at tapping into contextual characteristics of violence episodes and not necessarily scoping “minor” forms of violence, its context and underlying motivations [44]. This result points to the need of using standardized tools for IPV assessment (notably, the WHO tool and the CTS2 are the most frequent choices), to maximize comparability. Depending on the study objectives, there may also be a need to complement the use of such a standardized tool with other elements (questions or tools) that contextualize the violence experiences of interest.

Comparable direct quantifications of increased/decreased frequency of different types of IPV, that would allow pooling a summary measure for a change during the pandemic period, were not possible to retrieve from the studies included due to the heterogeneity in time periods assessed and pre-post comparisons established. However, we extracted narrative descriptions of changes provided by the included studies, and counted 22 studies indicating an increase, 9 suggesting a decrease and 11 that found either no-change compared to pre-pandemic frequencies or a mixture of increases and decreases in different types of violent experiences reported (or, for e.g., calls to helplines for homologous periods)—detailed in Supplementary Material Table S3.

### Strengths and limitations

This review summarized studies that assessed different forms of IPV during the Covid-19 pandemic, while trying to depict how methodological features may influence the prevalence estimates obtained. A strength of this study lies in the broad inclusion criteria, which allowed to aggregate studies on IPV not only against women, but also against men, for which there is less published evidence.

The attention paid, and criteria used, regarding the victim-perpetrator relationship (i.e., intimate partners), allowing to clearly identify and include only cases of IPV, rather than, for instance, domestic violence exerted by perpetrators other than intimate partners, is a strength of this study. Furthermore, a common issue in the field lies in the lack of use of clearly defined concepts, hindering comparison between studies. The inclusion of studies that identified IPV, regardless of the terms employed to refer to the phenomenon (such as partner abuse, battered women/men, domestic violence, etc., which were included as search terms in the present review) contributes to maximizing comparability to other work on the topic, by trying to use a harmonised definition. This harmonisation attempt also tried to ensure greater sensitiveness of the search and inclusion of relevant studies, maximizing “representativeness” and validity of IPV assessment during the pandemic.

The different methodological approaches taken in the studies included were categorized into fewer, meaningful categories. Some necessary simplifications might have impacted disclosure rates in different ways. For example, we did not distinguish studies aggregating across multiple standardized instruments (or using just one module or set of items of a specific standardized tool) from studies using complete versions of a single instrument.

We did not compare prevalence estimates within each of the categories of methodological features explored, according to further methodological features. Therefore, we cannot rule out the influence of the combination of these features on the frequency of violence. Future studies should try to explore if, for example, the prevalence found in studies using face-to-face contacts (compared to online), relates to other methodological features, such as type of instrument or sample.

Regarding face-to-face modes of administration, we did not collect information about sex or training of interviewers, which could also impact IPV disclosure rates.

The clinical samples included in the present review are a highly heterogeneous population; For example, they include pregnant women but also patients attending trauma clinics or emergency departments. Moreover, disclosure likely vary in terms of age, availability, and reasons to go to a healthcare service, which were characteristics not assessed, but that could impact disclosure of IPV. Among the studies resorting to clinical samples (*n* = 18), we noted that 11 conducted interviews with participants as described in their methods, while 7 were retrospective analysis of clinical records for the period considered. Future studies should also consider the potential influence of this methodological approach, which may impact the frequency of violence experiences documented in studies exploring clinical samples.

We did not consider the impact of geographic region, where the study was conducted, but provide estimates restricted to studies from USA as supplementary material, since we identified a total of 30 studies from this region. Also, we did not investigate IPV prevalence estimates disaggregated according to hetero- or non-heterosexual relationships, which could have provided further insights about potential influences of IPV during the pandemic.

Most of the studies included explored experiences of violence occurring during the pandemic or lockdown periods (*n* = 44), while the remaining studies used different referral periods (e.g., 13 studies mentioned a 12-month period, 11 studies described a 3-month period which largely overlaps the pandemic period, three studies refer to a 6-month period and at least two studies to the lifetime period – Supplementary material, Table S2). Although most of the studies in our review explored the pandemic period, it is important to note that the recall period can significantly impact prevalence estimates. Future studies attempting to summarise the influence of these features on IPV frequency should, therefore, consider different recall periods as another methodological feature to be explored.

The sensitivity analysis conducted with studies appraised as of high quality (Supplementary material, Figures S3.1 to S3.9), confirmed the overall results and conclusions drawn.

Finally, some of the funnel plots revealed asymmetry, suggesting a potential for publication bias among the included studies, despite our efforts to search multiple databases. The results, therefore, need to be interpreted cautiously.

## Conclusions

During the pandemic, published evidence until 2021 on IPV prevalence showed great variation in terms of the methodological choices and the estimates obtained. Most studies included in this review were conducted online, reflecting the required adaptation to lockdown restrictions imposed. The prevalence estimates observed were generally higher in face-to-face studies, and in studies using a standardized tool for IPV assessment. Despite the variation observed according to the methodological choices, the pooled estimates of the different forms of IPV found during the pandemic do not suggest marked changes in magnitude, when considering published global prevalence estimates from before COVID-19. Analysing the studies narratively, we also found more studies that suggested an increase in violence experiences during the pandemic, compared to studies describing a decrease, no-change, or a mixture of increased and decreased frequencies according to different violent behaviours. Nevertheless, future research on IPV should consider the impact of the methodological choices taken and try to use standardized tools and common definitions and, if possible, face-to-face assessments, to enhance comparability of their findings. There is also a need to explore other methodological characteristics that may impact violence frequencies, (such as the use of different recall periods), and assess the impact of combinations of the methodological features explored (e.g. influence of type of instrument and type of sample within studies conducted online or within studies using face-to-face mode of administration, or comparing estimates from studies exploring clinical records vs. interviews within studies assessing clinical samples). Finally, there is a need for more IPV research among samples of men. IPV remains a major global public health concern that must be tackled.

### Supplementary Information

**Additional file 1: Table S1.** Search results. **Figure S1.** Forest plot of physical intimate partner violence against women prevalence pooled by method of administration in telephone, online, face-to-face, or other. **Figure S2.** Forest plot of psychological intimate partner violence against women prevalence pooled by method of administration in telephone, online, face-to-face, or other. **Figure S3.** Forest plot of sexual intimate partner violence against women prevalence pooled by method of administration in telephone, online, face-to-face, or other. **Figure S4.** Forest plot of physical intimate partner violence against women prevalence pooled by type of sample in clinical, convenience, general population or community sample. **Figure S5.** Forest plot of psychological intimate partner violence against women prevalence pooled by type of sample in clinical, convenience, general population or community sample. **Figure S6.** Forest plot of sexual intimate partner violence against women prevalence pooled by type of sample in clinical, convenience, general population or community sample. **Figure S7.** Forest plot of physical intimate partner violence against women prevalence pooled by instrument used for assessment in standardized tool, specifically created questions or other. **Figure S8.** Forest plot of psychological intimate partner violence against women prevalence pooled by instrument used for assessment in standardized tool, specifically created questions or other. **Figure S9.** Forest plot of sexual intimate partner violence against women prevalence pooled by instrument used for assessment in standardized tool, specifically created questions or other. **Figures S10.** Funnel plots for all models. **Table S2.** Selected methodological details of the studies included. **Table S3.** Studies reporting changes in intimate partner violence frequency during the COVID-19 pandemic. **Table S4.** Quality Appraisal scores (Joanna Briggs Institute Tools).

**Additional file 2: Figure S2.1.** Forest plot of (“any” type of) intimate partner violence against women (VAW) prevalence pooled by method of administration (only “online” observed), between-study variance τ^2^= 0.00601417. **Figure S2.2.** Forest plot of (“any” type of) intimate partner violence against men (VAM) prevalence pooled by method of administration (only “online” observed), between-study variance τ^2^= 0.00621301. **Figure S2.3.** Forest plot of (“any” type of) intimate partner violence against women & men (VAM) prevalence pooled by method of administration in online and other. **Figure S2.4.** Forest plot of (“any” type of) intimate partner violence against women (VAW) prevalence pooled by type of sample in clinical, convenience or general population/community sample, between-study variance, τ^2^= 0.00601417. **Figure S2.5.** Forest plot of (“any” type of) intimate partner violence against men (VAM) prevalence pooled by type of sample (only “convenience” observed), between-study variance, τ^2^= 0.00621301. **Figure S2.6.** Forest plot of (“any” type of) intimate partner violence against women and men prevalence pooled by type of sample (only “convenience” observed). **Figure S2.7.** Forest plot of (“any” type of) intimate partner violence against women prevalence pooled by instrument used for assessment in standardized tool, specifically created questions, between-study variance, τ2= 0.00601417. **Figure S2.8.** Forest plot of (“any” type of) intimate partner violence against men prevalence pooled by instrument used for assessment in standardized tool and specifically created questions, between-study variance, τ^2^=0.00621301. **Figure S2.9.** Forest plot of (“any” type of) intimate partner violence against women and men prevalence pooled by instrument used for assessment in standardized tool and specifically created questions (from studies where sex-disaggregated prevalence estimates were not available).

**Additional file 3. Figure S3.1.** Forest plot of (“any” type of) intimate partner violence against women (VAW) prevalence pooled by method of administration, between-study variance τ^2^= 0.00552919. **Figure S3.2.** Forest plot of (“any” type of) intimate partner violence against men (VAM) prevalence pooled by method of administration (only “online” observed), between-study variance τ^2^= 0.00729038. **Figure S3.3.** Forest plot of (“any” type of) intimate partner violence against women & men (VAM) prevalence pooled by method of administration in online and other, between-study variance τ^2^= .09240205. **Figure S3.4.** Forest plot of (“any” type of) intimate partner violence against women (VAW) prevalence pooled by type of sample in clinical, convenience or general population/community sample, between-study variance, τ^2^= 0.00552919. **Figure S3.5.** Forest plot of (“any” type of) intimate partner violence against men (VAM) prevalence pooled by type of sample (only “convenience” observed), between-study variance, τ^2^= 0.00729038. **Figure S3.6.** Forest plot of (“any” type of) intimate partner violence against women and men prevalence pooled by type of sample (only “convenience” observed), between-study variance, τ2= .09240205. **Figure S3.7.** Forest plot of (“any” type of) intimate partner violence against women prevalence pooled by instrument used for assessment in standardized tool, specifically created questions, between-study variance, τ2= 0.00552919. **Figure S3.8.** Forest plot of (“any” type of) intimate partner violence against men prevalence pooled by instrument used for assessment in standardized tool and specifically created questions, between-study variance, τ^2^= 0.00729038. **Figure S3.9.** Forest plot of (“any” type of) intimate partner violence against women and men prevalence pooled by instrument used for assessment in standardized tool and specifically created questions (from studies where sex-disaggregated prevalence estimates were not available), between-study variance, τ^2^= 0.09240205.

## Data Availability

Detailed review lists can be provided by the corresponding author upon reasonable request.

## Abbreviations

IPV: Intimate partner violence
DV: Domestic violence
WHO: World Health Organization
95%CI: 95% Confidence Interval
VAW: Violence against women
VAM: Violence against men
CAS-SF: Composite abuse scale Short form
DVAWS: Domestic Violence Against Women Scale
VES: The Violence Exposure Scale
J-IPV: Jellinek inventory for assessing partner violence
CUSI: Coping Using Sex Inventory
CPS: Conflicts and Problems-Solving Scales
DVQ: Domestic Violence Questionnaire
ABI-R2: Abusive Behavior Inventory-R2
HITS: Hurt-Insult-Threaten-Scream
EPII: Epidemic–Pandemic Impacts Inventory
CARS: Cyber Aggression in Relationships Scale
CDAQ: Cyber Dating Abuse Questionnaire
