# Supplementary material for: Intimate partner violence during COVID-19: systematic review and meta-analysis according to methodological choices
Source: BMC Public Health. 2024 Jan 29;24:313. doi: 10.1186/s12889-024-17802-9 (PMC10823599; doi:10.1186/s12889-024-17802-9)
Supplement: Supplementary file 1 — Additional file 1: Table S1. Search results. Figure S1. Forest plot of physical intimate partner violence against women prevalence pooled by method of administration in telephone, online, face-to-face, or other. Figure S2. Forest plot of psychological intimate partner violence against women prevalence pooled by method of administration in telephone, online, face-to-face, or other. Figure S3. Forest plot of sexual intimate partner violence against women prevalence pooled by method of administration in telephone, online, face-to-face, or other. Figure S4. Forest plot of physical intimate partner violence against women prevalence pooled by type of sample in clinical, convenience, general population or community sample. Figure S5. Forest plot of psychological intimate partner violence against women prevalence pooled by type of sample in clinical, convenience, general population or community sample. Figure S6. Forest plot of sexual intimate partner violence against women prevalence pooled by type of sample in clinical, convenience, general population or community sample. Figure S7. Forest plot of physical intimate partner violence against women prevalence pooled by instrument used for assessment in standardized tool, specifically created questions or other. Figure S8. Forest plot of psychological intimate partner violence against women prevalence pooled by instrument used for assessment in standardized tool, specifically created questions or other. Figure S9. Forest plot of sexual intimate partner violence against women prevalence pooled by instrument used for assessment in standardized tool, specifically created questions or other. Figures S10. Funnel plots for all models. Table S2. Selected methodological details of the studies included. Table S3. Studies reporting changes in intimate partner violence frequency during the COVID-19 pandemic. Table S4. Quality Appraisal scores (Joanna Briggs Institute Tools). [file 12889_2024_17802_MOESM1_ESM.docx]

**Supplementary material**

Table S1. Search results

**MEDLINE via PubMed: 09.12.2021**

| **ID** | **Query** | **Results** |
| --- | --- | --- |
| #5 | #3 AND #4 | 690 |
| #4 | domestic violence[MeSH Terms] OR sex offenses[MeSH Terms] OR spouse abuse[MeSH Terms] OR spousal violence[MeSH Terms] OR relationship violence[MeSH Terms] OR couple violence[MeSH Terms] OR marital violence[MeSH Terms] OR intimate partner[All Fields] OR aggression[MeSH Terms] OR intimate partner violence[All Fields] OR physical violence[All Fields] OR emotional violence[All Fields] OR psychological violence[All Fields] OR sexual violence[All Fields] | 128,921 |
| **#3** | **#1 OR #2** | **218,435** |
| #2 | ("corona virus"[tw] OR "corona viruses"[tw] OR coronavir*[tw] OR coronovirus*[tw] OR betacoronavirus*[tw]) AND (novel[tw] OR 2019[tw] OR Wuhan[tw] OR Huanan[tw] OR Hubei[tw]) OR "new coronavirus"[tw] OR "COVID-19"[tw] OR COVID19[tw] OR "SARS coronavirus 2"[tw] OR "severe acute respiratory syndrome coronavirus 2"[tw] OR nCoV[tw] OR 2019nCoV[tw] OR nCoV2019[tw] OR "SARS-CoV-2"[tw] OR "SARS-CoV2"[tw] OR SARSCoV19[tw] OR SARS-CoV19[tw] OR SARS-CoV-19[tw] OR HCoV-19[tw] | 204,177 |
| #1 | "Coronavirus Infections"[Mesh] OR "Coronavirus"[Mesh] | 139,781 |

**MEDLINE via Web of Science Core Collection: 09.12.2021**

| **ID** | **Query** | **Results** |
| --- | --- | --- |
| #8 | #1 AND #6 Timespan=2019-12-30 – 2021-12-30 | 1,348 |
| #7 | #1 AND #6 | 1,358 |
| #6 | #4 OR #5 | 260,833 |
| #5 | TS= (domestic violence OR sex offenses OR spouse abuse OR spousal violence OR relationship violence OR couple violence OR marital violence OR intimate partner OR aggression OR intimate partner violence OR physical violence OR emotional violence OR psychological violence OR sexual violence) | 140,016 |
| #4 | #2 AND #3 | 198,458 |
| #3 | TS= (violence OR abuse OR batter* OR agress*) | 597,940 |
| #2 | TS= (domestic* OR intimate* OR spous* OR relation* OR relationship* OR family* OR couple* OR marital* OR physical* OR psychol* OR sexual* OR men OR women) | 7,956,863 |
| #1 | TS= (("corona virus" OR "corona viruses" OR coronavir* OR coronovirus* OR betacoronavirus*) AND (novel OR 2019 OR Wuhan OR Huanan OR Hubei) OR "new coronavirus" OR "COVID-19" OR COVID19 OR "SARS coronavirus 2" OR "severe acute respiratory syndrome coronavirus 2" OR nCoV OR 2019nCoV OR nCoV2019 OR "SARS-CoV-2" OR "SARS-CoV2" OR SARSCoV19 OR SARS-CoV19 OR SARS-CoV-19 OR HCoV-19 OR “corona vir*” OR “coronavir*” OR “betacoronavir*” OR “severe acute respiratory syndrome coronavirus”) | 236,204 |

**WHO COVID-19 database: 09.12.2021**

| **ID** | **Query** | **Results** |
| --- | --- | --- |
| #1 | (tw: (domestic* OR intimate* OR spous* OR relation* OR relationship* OR family* OR couple* OR marital* OR physical* OR psychol* OR sexual* OR men OR women) AND (violence OR abuse OR batter* OR agress*)) OR domestic violence OR sex offenses OR spouse abuse OR spousal violence OR relationship violence OR couple violence OR marital violence OR intimate partner OR aggression OR intimate partner violence OR physical violence OR emotional violence OR psychological violence OR sexual violence | 269 |

**The Cochrane Library: 09.12.2021**

| **ID** | **Query** | **Results** |
| --- | --- | --- |
| #16 | Of which “Trials” (NOT Reviews OR Editorials) | 1657 |
| #15 | #6 AND #14 time limit: Dec 2019 – Dec 2021 | 1673 |
| #14 | #6 AND #14 | 1681 |
| #13 | #7 OR #8 OR #9 OR #10 OR #11 OR #12 OR #13 | 346321 |
| #12 | (domestic*:ti,ab,kw OR intimate*:ti,ab,kw OR spous*:ti,ab,kw OR relation*:ti,ab,kw OR relationship:ti,ab,kw OR family:ti,ab,kw OR couple:ti,ab,kw OR marital:ti,ab,kw OR physical*:ti,ab,kw OR psychol*:ti,ab,kw OR sexual*:ti,ab,kw  AND (violence:ti,ab,kw OR abuse:ti,ab,kw OR batter*:ti,ab,kw OR agress*:ti,ab,kw)) OR “domestic violence”:ti,ab,kw OR “sex offenses”:ti,ab,kw OR “spouse abuse”:ti,ab,kw OR “spousal violence”:ti,ab,kw OR “relationship violence”:ti,ab,kw OR “couple violence”:ti,ab,kw OR “marital violence”:ti,ab,kw OR “intimate partner”:ti,ab,kw OR “aggression”:ti,ab,kw OR “intimate partner violence”:ti,ab,kw OR “physical violence”:ti,ab,kw OR “emotional violence”:ti,ab,kw OR “psychological violence”:ti,ab,kw OR “sexual violence”:ti,ab,kw | 346287 |
| #11 | MeSH descriptor: [Gender-Based Violence] explode all trees | 9 |
| #10 | MeSH descriptor: [Family Conflict] explode all trees | 99 |
| #9 | MeSH descriptor: [Spouse Abuse] explode all trees | 199 |
| #8 | MeSH descriptor: [Intimate Partner Violence] explode all trees | 395 |
| #7 | MeSH descriptor: [Domestic Violence] explode all trees | 893 |
| #6 | #1 OR #2 OR #3 OR #4 #5 | 8504 |
| #5 | ("corona virus":ti,ab,kw OR "corona viruses":ti,ab,kw OR coronavir*:ti,ab,kw OR coronovirus*:ti,ab,kw OR betacoronavirus*:ti,ab,kw) AND (novel:ti,ab,kw OR 2019:ti,ab,kw OR Wuhan:ti,ab,kw OR Huanan:ti,ab,kw OR Hubei:ti,ab,kw) OR "new coronavirus":ti,ab,kw OR "COVID-19":ti,ab,kw OR COVID19:ti,ab,kw OR "SARS coronavirus 2":ti,ab,kw OR "severe acute respiratory syndrome coronavirus 2":ti,ab,kw OR nCoV:ti,ab,kw OR 2019nCoV:ti,ab,kw OR nCoV2019:ti,ab,kw OR "SARS-CoV-2":ti,ab,kw OR "SARS-CoV2":ti,ab,kw OR SARSCoV19:ti,ab,kw OR SARS-CoV19:ti,ab,kw OR SARS-CoV-19:ti,ab,kw OR HCoV-19:ti,ab,kw OR WN-CoV:ti,ab,kw | 8458 |
| #4 | MeSH descriptor: [SARS-CoV-2] explode all trees | 599 |
| #3 | MeSH descriptor: [COVID-19] explode all trees | 918 |
| #2 | MeSH descriptor: [Coronavirus] explode all trees | 612 |
| #1 | MeSH descriptor: [Coronavirus Infections] explode all trees | 1463 |

**PsychInfo via EBSCO: 09.12.2021**

| **ID** | **Query** | **Results** |
| --- | --- | --- |
| #4 | #1 AND #2 Limiter Date: 20191201-20211231 | 244 |
| #3 | #1 AND #2 | 297 |
| #2 | domestic violence OR sex offenses OR spouse abuse OR spousal violence OR relationship violence OR couple violence OR marital violence OR intimate partner OR aggression OR intimate partner violence OR physical violence OR emotional violence OR psychological violence OR sexual violence | 137,169 |
| #1 | corona virus OR corona viruses OR coronavir OR coronovirus OR betacoronavirus  OR Wuhan OR Huanan OR Hubei OR new coronavirus OR COVID-19 OR COVID19 OR SARS coronavirus 2 OR severe acute respiratory syndrome coronavirus 2 OR nCoV OR 2019nCoV OR nCoV2019 OR SARS-CoV-2 OR SARS-CoV2 OR SARSCoV19 OR SARS-CoV19 OR SARS-CoV-19 OR HCoV-19 OR WN-CoV | 16,830 |

**CINAHL via EBSCO: 09.12.2021**

| **ID** | **Query** | **Results** |
| --- | --- | --- |
| #4 | #1 AND #2 Limiter Date: 20191201-20211231 | 339 |
| #3 | #1 AND #2 | 345 |
| #2 | domestic violence OR sex offenses OR spouse abuse OR spousal violence OR relationship violence OR couple violence OR marital violence OR intimate partner OR aggression OR intimate partner violence OR physical violence OR emotional violence OR psychological violence OR sexual violence | 51,376 |
| #1 | corona virus OR corona viruses OR coronavir OR coronovirus OR betacoronavirus  OR Wuhan OR Huanan OR Hubei OR new coronavirus OR COVID-19 OR COVID19 OR SARS coronavirus 2 OR severe acute respiratory syndrome coronavirus 2 OR nCoV OR 2019nCoV OR nCoV2019 OR SARS-CoV-2 OR SARS-CoV2 OR SARSCoV19 OR SARS-CoV19 OR SARS-CoV-19 OR HCoV-19 OR WN-CoV | 78,497 |

Figure S1. Forest plot of physical intimate partner violence against women prevalence pooled by method of administration in telephone, online, face-to-face, or other.

Figure S2. Forest plot of psychological intimate partner violence against women prevalence pooled by method of administration in telephone, online, face-to-face, or other.

Figure S3. Forest plot of sexual intimate partner violence against women prevalence pooled by method of administration in telephone, online, face-to-face, or other.

Figure S4. Forest plot of physical intimate partner violence against women prevalence pooled by type of sample in clinical, convenience, general population or community sample.

Figure S5. Forest plot of psychological intimate partner violence against women prevalence pooled by type of sample in clinical, convenience, general population or community sample.

Figure S6. Forest plot of sexual intimate partner violence against women prevalence pooled by type of sample in clinical, convenience, general population or community sample.

Figure S7. Forest plot of physical intimate partner violence against women prevalence pooled by instrument used for assessment in standardized tool, specifically created questions or other

Figure S8. Forest plot of psychological intimate partner violence against women prevalence pooled by instrument used for assessment in standardized tool, specifically created questions or other

Figure S9. Forest plot of sexual intimate partner violence against women prevalence pooled by instrument used for assessment in standardized tool, specifically created questions or other

Figures S10. Funnel plots for all models


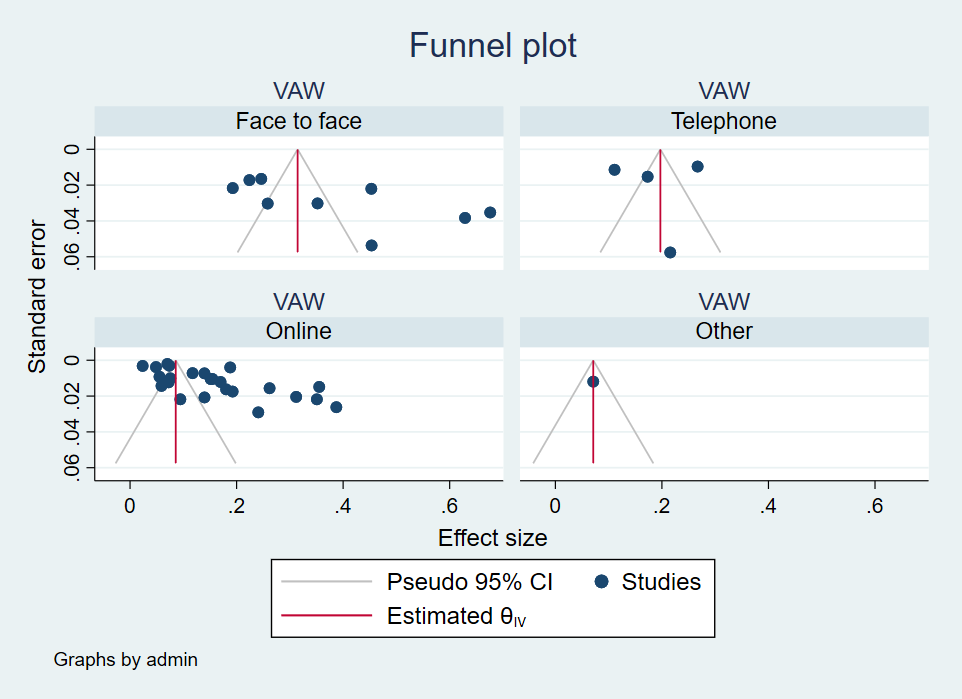


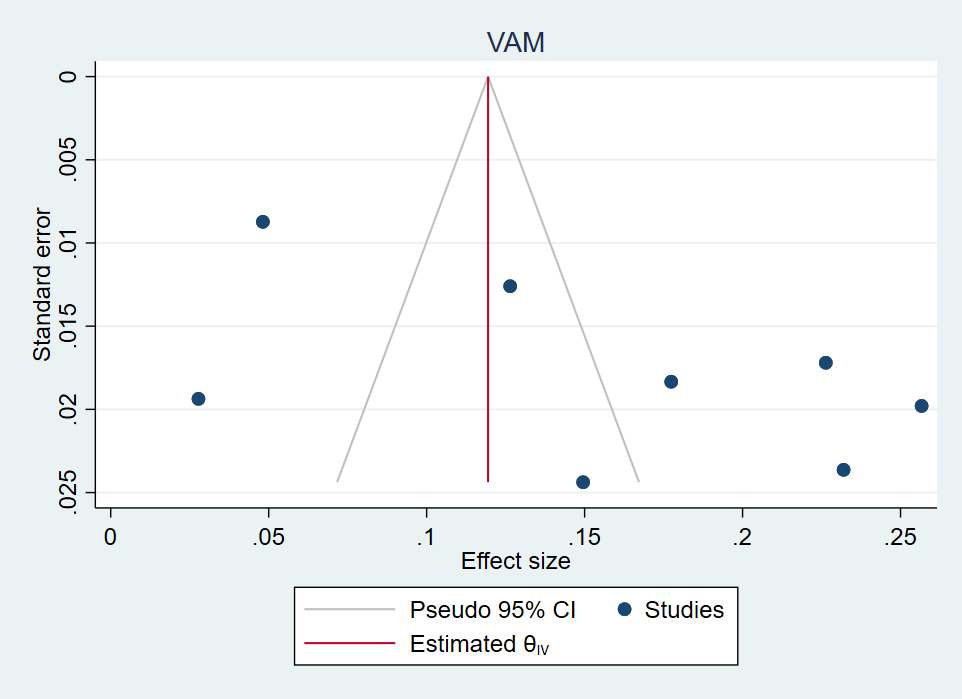


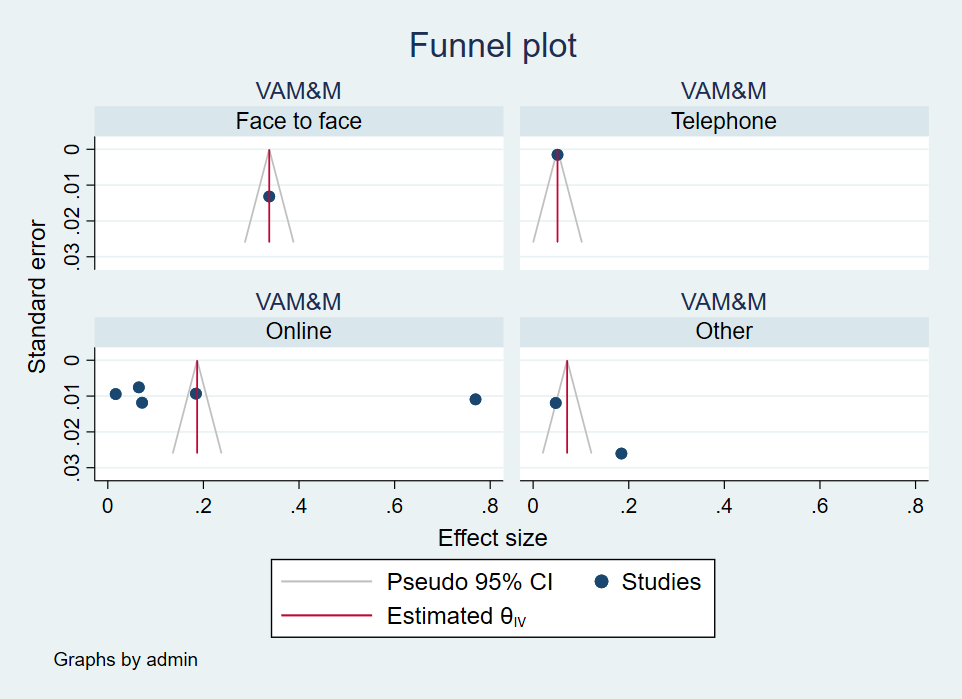

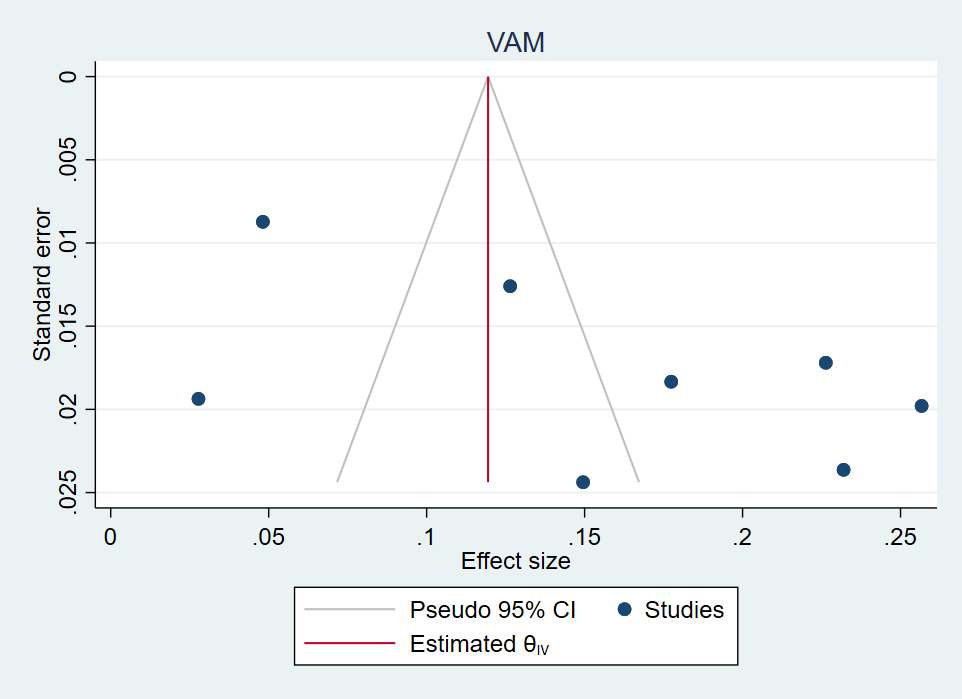

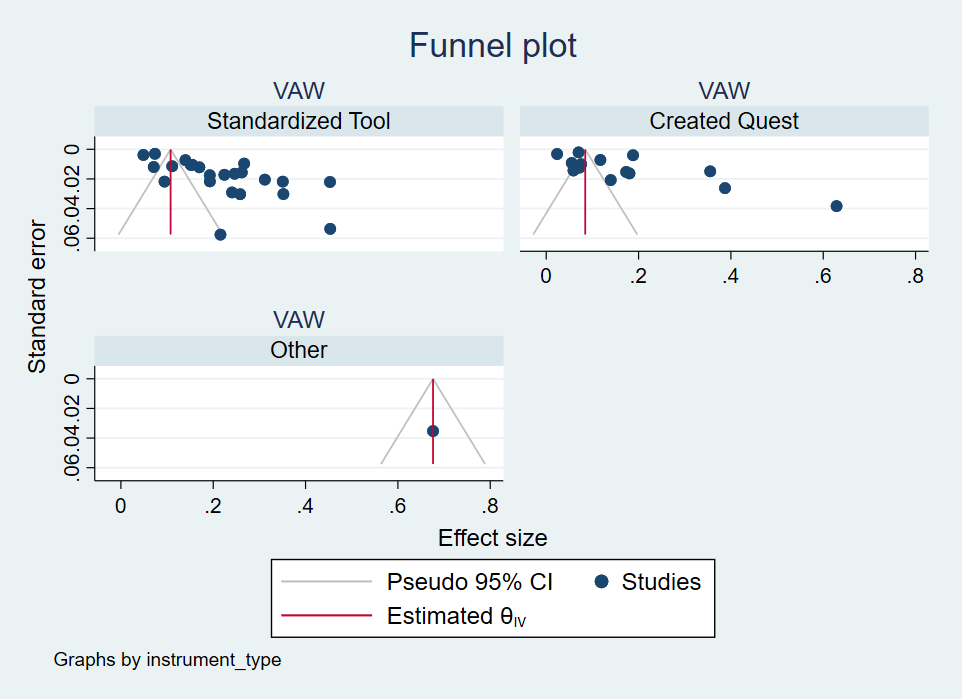

Table S2. Selected methodological details of the studies included.

| **Author, year** | **Country/region** | **Sample size Total** | **Violence against Women (VAW), men (VAM), or both (VAW&M)** | **Type of sample (general population/community, clinical, victims, students, other convenience)** | **IPV Tool/instrument/questionnaire (or adaptation of validated tool)** | **Referent period of IPV questions/experiences** |
| --- | --- | --- | --- | --- | --- | --- |
| Abrahams,2020 | South Africa | 885 | VAW | Clinical (perinatal women) | The CAS-SF - Composite abuse scale (revised) - short form | Past 12 months |
| Abuhammad,2020 | Jordan | 687 | VAW | Convenience recruited online | NA | NA |
| Abujilban,2021 | Jordan | 215 | VAW | pregnant women convenience sample | Arabic version of the WHO: Domestic Violence Questionnaire Screening Tool (DVQST) | women were asked to respond to the psychological, physical, and sexual IPV items twice, one time for “before quarantine” and one time for “during quarantine” |
| Adibelli,2021 | Turkey | 332 | VAW | community | Domestic Violence Against Women Scale (DVAWS) | NA |
| Aguero,2020 | Peru | 1020 | VAW | helpline callers | NA | NA |
| Akalin,2021 | Turkey | 1036 | VAW | Convenience recruited online | NA | The women were asked about each act of violence, and whether it had occurred during the COVID-19 pandemic (as of March 16, 2020), and then about the frequency of each act |
| Akel,2020 | Lebanon | 172 | VAW&M | Randomly selected convenience sample | The CAS-SF - Composite abuse scale (revised) - short form | NA |
| Alharbi,2021 | Saudi Arabia | 2254 | VAW | Convenience recruited online through social media | Arabic version of the World Health Organization (WHO) multi-country instrument related to violence against women | NA |
| Arenas-Arroyo,2020 | Spain | 8951 | VAW | Community | Included nine different situations, that were obtained from a larger set of situations in the last Survey on Violence Against Women in Spain | Before and during the lock-down in Spain |
| Artinopoulou,2021 | Greece | 705 | VAW | community | NA | Before and during the first lockdown in Greece from March to May 2020 |
| Barbara,2020 | Italy |  | VAW | Victims | NA | Study period |
| Barchielli,2021 | Italy | 211 | VAW&M | Convenience (newspaper reports | NA | Study period |
| Bhattaram,2021 | India | 828 | VAW | Clinical | NA | Study period |
| Boman,2020 | USA |  | VAW&M | Convenience | NA | Study period |
| Bourne,2021 | Jamaica | 513 | VAW | Community | The instrument was an adaptation of one developed by Jones (2021) to assess stress among Jamaicans. | NA |
| Boxall,2021 | Australia | 9284 | VAW | Convenience | Psychological Maltreatment of Women Inventory–Short Form’s Dominance–Isolation subscale (Tolman 1999). Other items relating to emotional abuse and stalking were based on the Personal Safety Survey (ABS 2017), with the addition of a question about technology-facilitated abuse. | 3 months prior to survey |
| Bueso-Izquierdo,2021 | Spain | 155 | VAW | Convenience | NA | Lifetime |
| Bullinger,2021 | USA |  | VAW&M | general population | NA | NA |
| Buttell,2021 | USA | 374 | VAW&M | Convenience | NA | Lifetime |
| Campedelli,2020 | USA |  | VAW&M | general population | NA | NA |
| Cannon,2021 | USA | 374 | VAW&M | convenience sample | NA | During the pandemic |
| Cano-Lozano,2021 | Spain | 2245 | VAW | Convenience | The Violence Exposure Scale (VES) - Domestic violence subscale | Lifetime |
| Capinha,2021 | Portugal |  | VAW&M |  | NA | NA |
| Chen,2021 | USA | 222 | VAW&M | Black men who have sex with men (BMSM, n = 196) and transgender women (BTW, n = 20). In the Chicago metropolitan area. (6 reported other as gender.) | NA | since shelter-in-place order (March 21, 2020) - includes the assessment period when the order was not in place anymore: Illinois reopening phases, were June 2 and before represents the “shelter-in-place” order period and phase 1 (March 21, 2020–April 30, 2020)/phase 2 (May 1, 2020–June 2, 2020) and June 3 and after represents phase 3 (June 3, 2020–June 25, 2020)/phase 4 (June 26, 2020 and after) |
| Clemens,2021 | Germany | 687 | VAW&M | parents of underaged children in Germany | NA | in the relationship |
| Daigle,2021 | USA | 13,373 | VAW&M | The data include survey responses from 13,373 students attending 22 diferent institutions of higher learning. | NA | within the past 12 months |
| Das,2021 | India | 159 | VAW | Community | NA | Two months preceding the survey |
| Davis,2021 | USA | 2045 | VAW&M | Convenience | Jellinek inventory for assessing partner violence (J-IPV) | Since the beginning of the pandemic |
| Decker,2021 | Kenya | 1217 | VAW&M | Convenience, An existing cohort of youth ages 16–26 in Nairobi, Kenya | NA | past-year |
| Dekel,2021 | South Africa | 16 | VAW |  | NA | NA |
| Di Franco,2020 | Italy | 19160 | VAW&M | clinical - attending emergency room | The self-administered questionnaire of the WHO Multi-country Study on Women’s Health and Domestic Violence against Women | period between 1 January and 2 June 2020 |
| Ditekemena,2021 | Congo | 2002 | VAW | convenience recruited online via social media platforms | NA | IPV during the confinement period (March through June 2020) |
| Ebert,2021 | Germany | 3818 | VAW | representative of partnered women | Small number of questions rather than the full WHO domestic violence questionnaire. Adapted measures of violence from previous surveys conducted in Germany. | during the previous month |
| El-Nimr,2021 | Arab women - different countries | 490 | VAW | convenience online | NA | exposure to different types of IPV before and during COVID-19 lockdown |
| Erausquin,2021 | Multicountry | 22724 | VAW&M | 23 studies used convenience sampling, six studies used online panel, and two used population-based methods | NA | NA |
| Farmani,2020 | Iran | 20885 | VAW | Fars Province, Iran; The sample of this study was cases of psychosocial disturbances registered in the health system of Social Emergency Centers of Fars Province (SECFP) on a monthly basis to the provincial social emergency center. All they called emergency centres for receiving help from mental health professionals at SECFP. | for young women, past-year experience of intimate partner violence (IPV), non-partner sexual violence, and timing of IPV experiences relative to COVID-19 restrictions were assessed via best practices for violence assessment | February 20 to May 21, 2020 (during the COVID-19 Pandemic) |
| Fereidooni,2021 | Iran | 2116 | VAW | community random sample (random digit dialing) | WHO tool - Persian version | The primary outcome was self-reported experience of IPV during the last six months |
| Fleming,2021 | USA | 782 | VAW | convenience online sample | Relationship conflict was measured using two items from the Conflict Tactics Scale-2 Short Form (CTS-2 Short Form) measuring verbal aggression. The CTS-2 Short Form is a 20-item measure evaluating various types of relationship conflict techniques, and items from the verbal aggression subscale were used in this study. | NA |
| Gama,2021 | Portugal | 1062 | VAW&M | online convenience sample | The questionnaire was developed by Ghent University based on the UN-MENAMAIS Study questionnaire, drawn on a set of validated instruments. Minor adaptations were made to the national context in terms of language and the response options to some questions (e.g., level of education). | reported experience of domestic violence during the COVID-19 pandemic |
| Gebrewahd,2020 | Ethiopia | 682 | VAW | community sample | A validated structured questionnaire, adopted from WHO core questionnaire on domestic intimate partner violence, was prepared in the local language (Tigrigna). The result on violence against women was obtained using thirteen WHO questions regarding psychological, physical and sexual violence. | during COVID-19 lockdown |
| Ghimire,2020 | Nepal | 556 | VAW&M | convenience online sample | Information regarding domestic violence and substance abuse were collected using questions designed by the study team consisting of medical doctors and psychiatrists. The questions were included on the basis of face validity | during the lockdown period |
| Gibbons,2021 | Argentina | 1502 | VAW | convenience online sample of women | IPV was a primary outcome measured using an adapted six-item version of the WHO IPV scale. | We asked for intimate partner violence in the one-year period before quarantine and since the beginning of the quarantine (a period of two months). |
| Gillespie,2020 | UK | 789 | VAW&M | Internet platform, convenience paid | Coping Using Sex Inventory (CUSI) | asked them to report on their use of sex as a means to cope over the last 14 days, and retrospectively over the 14-day period that preceded the introduction of government-enforced lockdown |
| Gleason,2021 | USA | 1051 | VAW&M | convenience online | NA | NA |
| Gosangi,2020 | USA | 62 | VAW | Clinical sample, radiology images of patients | NA | NA |
| Gresham,2021 | USA | 1813 | VAW&M | general population (convenience national health volunteer registry) | Experience with Battering and the 29-item Abuse Behavior Inventory | during pandemic |
| Hamadani,2020 | Bangladesh | 2424 | VAW | (mothers of ) singleton children without a diagnosed blood disorder or severe anaemia (Hb <80 g/L) were eligible when they were aged 8 months; community households selected. | WHO tool | since the last days of March 2020 |
| Helland,2021 | Norway | 1747 | VAW&M | clinical sample: parents attending parental counselling, therapy or mediation | Interparental conflicts were measured with the Verbal aggression (e.g., “Name-calling, cursing, insulting”), Physical aggression (e.g., “Throw something at the other”), and Child involvement (e.g., “Involve the child in our argument”) subscales from a short-version of the Conflicts and Problems-Solving Scales (CPS). | NA |
| Holland,2021 | USA | 187508065 | VAW&M | clinical: national records of ED utilization (public health surveillance data) | NA | NA |
| Indu PV,2021 | India | 209 | VAW | community sample of women | Domestic violence was defined as any form of physical, psychological or sexual violence perpetrated by husband towards a woman, and was measured using the Domestic Violence Questionnaire (DVQ) | NA |
| Jetelina,2020 | USA | 1730 | VAW&M | convenience online sample | IPV was measured using the validated 5-item, Extended Hurt, Insulted, Threated and Scream (E-HITS) construct | 1=never; 2=rarely; 3=sometimes; 4=fairly often; 5=frequently. COVID-19-related IPV severity: of the participants that screened positive for E-HITS, a follow-up question was asked: “Since the coronavirus outbreak, has this gotten… 1) Much better; 2) Somewhat better; 3) Stayed the same; 4) Somewhat worse; and 5) Much worse”. Response categories were further categorised into: (1) much better/ somewhat better; (2) stayed the same; and (3) somewhat worse/much worse. |
| Johnson,2021 | USA | 183 | VAW | non-probbility sample of pregnant women (convenience) | Psychological abuse was measured using the Psychological Abuse subscale from the Abusive Behavior Inventory-R2 (ABI-R2); Physical and sexual abuse were measured using questions adapted from the Two-Question Screening Tool. | Experiences (psychological, physical, sexual abuse) in the past 12 months |
| Karp C,2021 | Kenya | 756 | VAW | general population of girls/adolescents followed in the scope of established cohorts | experience of IPV (yes/no) based on reports of emotional, physical, or sexual violence perpetrated by the partner in the last month using a modified version of the Revised Conflict Tactics Scale | last month |
| Kimura M,2021 | Japan | 2286 | VAW | general pop: online surveys hosted by a survey company that had a nationwide research panel of 4.7 million | NA | Between March and May 2020 |
| Kliem,2021 | Germany | 1005 | VAW&M | general population (representative survey) | Family Maltreatment Measure | physical partner violence in the preceding 12 months |
| Krishnamurti,2021 | USA | 959 | VAW | clinical (preganant sample) | Two questions from the Centers for Disease Control and Prevention Behavioral Risk Factor Surveillance System as measures of physical violence and forced sexual acts. Also used 10 questions from the Women’s Experience with Battering scale to quantify psychological abuse | Past month items for Physical and Sexual and Women Experiences with Battering for Psychological |
| Lampe,2021 | Austria | 67 | VAW&M | clinical (general hospital patients) | adapted German version of the Hurt-Insult-Threaten-Scream (HITS) scale | last two weeks |
| Lee,2021 | USA | 291 | VAW&M | convenience online sample | NA | past two weeks |
| Lindau,2021 | USA | 3200 | VAW | online panel established to represent the US general pop | Centers for Medicare & Medicaid Services Accountable Health Communities (AHC) screening tool (includes the HITS for IPV) | prepandemic and early phase of pandemic (adapted from HITS) - past 12 months |
| Logie,2021 | Uganda | 367 | VAW&M | data collected as part of the Tushirikiane cluster randomized control trial among displaced and refugee adolescent youth in Kampala, Uganda (community) | Experiences of physical or sexual IPV were assessed using an adapted short form of the Conflict Tactics Scale as measured in the Ugandan Demographic and Health Surveys. | NA |
| MacCarthy,2020 | USA | 52 | VAW&M | (clinical) users of Bienestar, a community-based organization with six offices across LAC. Bienestar is focused on serving Latinx communities including HIV-positive people, individuals at risk for HIV, LGBT, youth and people who use substances. | based on the Epidemic–Pandemic Impacts Inventory [EPII], a tool designed to assess tangible impacts of COVID-19 across personal and social life domains - includes one item on IPV during quarantine | During pandemic |
| Maftei,2021 | Romania | 1113 | VAW&M | convenience recruited online (mostly in university students forums) | The dependent variable, technological intimate partner violence, was measured using the Cyber Aggression in Relationships Scale | technological intimate partner violence perpetration in the last six months and added the scale’s victimization-version |
| Mahmood,2021 | Iraq | 346 | VAW | convenience recruited online (mostly in university students forums) | based on previous literature, WHO (2000) and Garcia-Moreno et al (2005). | Asked about experiences 2 months preceding the lockdown, and the lockdown period, separately |
| Maxine,2020 | USA | 2045 | VAW&M | general population (recruited online via Qualtrics platform) | Jellinek inventory for assessing partner violence (J-IPV). The J-IPV is a 4-item screening tool developed to assess IPV victimization and perpetration in patients entering substance abuse treatment. Four-item tool was used to assess IPV perpetration and victimization since the outbreak of COVID-19. The rapid tool inquired about two forms of IPV, psychological and physical | Participants were asked about recent engagement or experience of IPV, e.g. : "since the coronavirus crisis started, the situation with your partner got so out of hand that you acted in a threatening way to your partner, or threatened to hurt him/her"? ‘Yes’=1; ‘No’=0. |
| McLean,2021 | Australia | 8435 | VAW&M | healthcare professionals, (convenience) | violence against a woman perpetrated by an intimate partner (5 items) - Questions were adapted from the DOORS family violence screening tool | frequency of incidents of family violence in their caseload, occurring during May 2020 |
| McMillan,2021 | USA | 49 | VAW | convenience | NA | NA |
| Moawad,2021 | Egypt | 509 | VAW | convenience recruited online (large facebook and whatsapp groups) | definitions of types and different forms of violence were adopted from the available literature Egypt demographic and health survey 2014 | "before and during the COVID-19 pandemic" - but for violence by any perpetrator |
| Morgan,2020 | Australia | 7446 | VAW | convenience online sample | The questions about physical violence were taken from the Personal Safety Survey. Respondents were also asked a question from the same source about sexual violence | asked respondents about their experience of domestic violence in the three months since February 2020 (The dependent variable in this study was whether the respondent had experienced physical or sexual violence by an intimate partner in the three months prior to the survey |
| Muldoon (1),2021 | Canada | 35 | VAW&M | clinical (ED patients triaged for DV) | NA | NA |
| Muldoon (2),2021 | Canada | 216 | VAW | clinical, post-partum women identified in hospital | Perinatal IPV was measured by two scales from the WHO multicountry study on domestic violence. Scale one, comprised of eight questions, measures different forms of regular controlling behaviour exhibited by the partner (eg, insist on knowing where you are at all times, try to keep you from seeing your friends, are often suspicious that you are unfaithful, etc). Scale two measures act-based forms of IPV including emotional abuse (ie, insulting, belittling, intimidating, threatening to hurt someone you care about), physical abuse (ie, slapped, pushed, hit, strangled, threatened with a weapon), and sexual abuse (ie, forced to have unwanted sexual intercourse, forced to have other unwanted sexual activities, forced to engage in unwanted sexual activities they considered degrading or humiliating) | Perinatal IPV was defined as regular controlling behaviours or act-based forms of emotional/physical/sexual abuse in the 12 months before pregnancy, during pregnancy and/or post partum. The act-based forms of perinatal IPV were asked for each perinatal time period: 12 months before pregnancy, during pregnancy and post partum. |
| Naghizadeh,2021 | Iran | 250 | VAW | convenience sample of pregnant women in the hospital | WHO questionnaire. In this questionnaire, physical, sexual, and emotional violence are assessed by 10, five, and 11 items, respectively | "During the Covid-19 outbreak" |
| Nyashanu,2021 | Zambia | 40 | VAW | convenience sample of women | NA | NA |
| Oginni,2021 | Nigeria | 966 | VAW&M | convenience sample of adults recruited online (social media) | HARK questionnaire which comprises four questions each screening for past-year physical, sexual and emotional abuse | Past year physical, sexual and emotional abuse |
| Ogrodniczuk,2021 | Canada | 434 | VAM | convenience sample of men recruited via website for helping with mental health symptoms | NA | "Since COVID-19" |
| Ojeahere,2021 | Nigeria | 474 | VAW&M | convenience sample (online, researchers network and snowball) | Adaptations of the computer based IPV questionnaire (Basile et al., 2007) | "Prior to lockdown" and "During the period of lockdown" |
| Olushola,2021 | Nigeria | 755 | VAW&M | convenience sample (online) | NA | "during the lockdown" |
| Overall,2021 | New Zealand | 155 | VAW&M | convenience sample of parents entering a previous longitudinal study, recruited via advertising in parenting magazines, early childhood centres and facebook (…) | eight-item verbal aggression subscale from Conflict and problem-solving scales | Parents then reported on their aggression toward partners and children during a nationwide lockdown in New Zealand (NZ), which involved mandatory and enforced confinement in the home for 5 weeks (March 26 to April 28, 2020) |
| Pal,2021 | India | 271 | VAW&M | convenience sample gathered online - social media | Composite abuse scale (revised short form) (CAS-SF) | "before and during" lockdown |
| Parrott,2021 | USA | 510 | VAW&M | convenience sample (non probability samplke recruited online) | Select items from the Psychological Aggression and Physical Aggression subscales of the Revised Conflict Tactics Scale assessed IPA perpetration. Psychological IPA was assessed with four items (i.e., “I yelled at my partner,” “I sulked or withdrew from my partner,” “I insulted or called my partner names,” “I made threats to my partner”) and physical IPA was assessed with two items (i.e., “I threw things, kicked, or hit something” and “I pushed, grabbed, or hit”). Participants rated the frequency of each item on 7-point scales | in the 6 months before SiP restrictions and in the period of time between the implementation of these restrictions and completing the survey |
| Pattojoshi,2020 | India | 560 | VAW | convenience sample (online via whatsapp groups) | NA | spousal violence experienced by Indian women during the lockdown |
| Peitzmeier,2021 | USA | 1169 | VAW | Population-based: qualtrics quota matched to population Census (age, education, income) | Physical (five items), sexual (three items), and psychological (seven items) IPV were measured with items drawn from the National Intimate Partner Violence and Sexual Violence survey (NISVS) and the Danger Assessment. The technology-facilitated IPV measure included four items drawn from the Cyber Aggression in Relationships Scale, and one developed based on Freed et al. | lifetime exposure to physical, sexual, psychological, or technology-based IPV using a total of 20 behavioral items. If participants reported any lifetime exposure, they were then asked to identify whether the behaviors occurred (1) in the 3 months since COVID began, as marked by when stay-at-home orders were first implemented (March 2020–June 2020); (2) in the 3 months prior to stay-at-home orders (December 2019–February 2020); and (3) sometime prior to December 2019. |
| Plasilova,2021 | Czech Republic | 429 | VAW | Population-based: based on quotas by external organisation | scales and questionnaires were selected based on topics agreed by the I-SHARE consortium. IPV: adapted shortened six-item questionnaire derived from the WHO IPV interview | women reported IPV incidents 3 months before and during the first and second waves of the COVID-19 pandemic. (1) three months before the COVID-19 pandemic (t0); (2) the first wave of the COVID-19 pandemic: 12 March 2020 to 17 May 2020 (t1); (3) the second wave of the COVID-19 pandemic: 5 October 2020 to the time of questionnaire completion (t2). They were asked to adjust their retrospective self-reported answers to these specific periods framed by the concrete dates that were characterized by strong restrictive measures in the CR. |
| Radusky,2021 | Argentina | 182 | VAW&M | convenience sample (online, specific groups) - ((72 transfeminine [TF], 66 transmasculine [TM], 44 non-binary [NB] people) | NA | "during lockdown" |
| Rahman,2021 | Egypt | 312 | VAW&M |  | NA | NA |
| Ravi,2021 | USA | 10 | VAW | convenience sample from a larger study (recruited in IPV shelters) | NA | NA |
| Rayhan,2021 | Bangladesh | 510 | VAW | convenience, community sample | translated version of WHO multi-country study tools to screen the IPV | participants were requested to share their experience of violence from 26 March (the time of the official closure of both government and private offices with restriction on mobility) to 26 August, on the basis of these 5 months |
| Rhodes,2020 | USA | 50 | VAW&M | clinical (ED patiens to level one trauma centre) |  |  |
| Rockowitz,2021 | Kenya | 317 | VAW&M | clinical (survivors accessing services) | NA | NA |
| Rodriguez,2021 | USA | 716 | VAW&M |  | Selected eight items from the shortened Conflict Tactics Scale—Short Form | last 2 weeks |
| Rojas-Solis,2021 | Mexico | 307 | VAW&M | university students convenience sample | Cyber Dating Abuse Questionnaire (CDAQ) | NA |
| Sabri,2021 | USA | 45 | VAW | convenience clinical (survivors) sample | NA | NA |
| Schokkenbroek,2021 | Belgium | 1491 | VAW&M | convenience community sample | Verbal partner violence. was measured with a short version of the aggression subscale of the Conflict and Problem Solving Scales | "how frequently various verbal altercations with their partner had occurred during the lockdown" |
| Spencer,2021 | USA | 365 | VAW&M | convenience community sample recrtuited via online platform for surveys | IPV perpetration was assessed using the adapted Universal Violence Prevention Screening Protocol | Participants were asked, within the past year, if they had engaged in IPV-related activities |
| Stephenson,2021 | USA | 696 | VAM | convenience community GBMSM recruited online | The recent experience of IPV was assessed using the gay, and bisexual men intimate partner violence (IPV-GBM) scale adapted by Stephenson from the Conflict Tactics scale to more accurately measure IPV among gay and bisexual men | analysis considers changes in victimization and perpetration of IPV during the 3 months prior to the survey (March-May 2020) "Recent experiences" |
| Tadesse,2020 | Ethiopia | 589 | VAW | general population sample (random) | WHO-2005 multi-country violence against women assessment tools and modified in contexts | Last three months |
| Takaku,2021 | Japan | 15836 | VAW | convineence, online mothers of children aged 4-10; but aiming for representativeness | NA | NA |
| Tesfaw,2021 | Ethiopia | 1288 | VAW&M | general population random sample | NA | sexual violence during the pandemic |
| Teshome,2021 | Ethiopia | 464 | VAW | clinical (pregnant women at clinic) | The WHO multi-country study on women's health and domestic violence against women questionnaire was used to assess IPV. | lifetime, year before interview ad during pregnancy |
| Tierolf,2020 | Netherlands | 87 | VAW&M | victims/clinical sample recruited in previous study of families helped through support agencies | Dutch translation of the Revised Conflict Tactics Scale-2 | past year IPV (all subscales of the CTS2) |
| Vives-Cases,2021 | Spain |  | VAW | calls, victims, registers | NA | The time periods considered to calculate the rates were from the first quarter (Q1) of 2015 up to the third quarter (Q3) of 2020; the second quarter (Q2) of 2020 was that affected by the COVID-19 induced lockdown (14 March to 21 June 2020). |
| Walsh,2021 | USA | 214 | VAM | convenience sample of gay, bisexual, and other men who have sex with men (GBMSM) - part of larger study | Gay and Bisexual Men Intimate Partner Violence scale adapted by Stephenson (for use in GBMSM populations) from the Conflict Tactics scale to reflect recent IPV experiences among GBMSM | this scale was used to ask men about their experiences of victimization and perpetration within their primary relationship, during the pandemic |
| Wong,2021 | China (Hong Kong) | 600 | VAW&M | community sample of parents recruited online (mobile phone list) | IPV was assessed by the Chinese version of the Abuse Assessment Screen | during Covid-19 (Jan to April 2020) |
| Yari,2021 | Iran | 203 | VAW | convenience recruited online | NA | The subjects were asked to complete the questionnaire based on their experiences during the COVID-19. |

Table S3. Studies reporting changes in intimate partner violence frequency during the COVID-19 pandemic.

| **Author, year** | **Country/region** | **Sample size Total** | **Violence against Women (VAW), men (VAM), or both (VAW&M)** | **Difference pre-post pandemic reported (description)** | **Increase/decrease reported (description)** |
| --- | --- | --- | --- | --- | --- |
| Abujilban,2021 | Jordan | 215 | VAW | Before the pandemic figures were: physical=30.7%(66), psychological=65.1%(140), sexual=15.3%(33) | **Decrease:** the results showed that the women experienced higher incidences of IPV before the quarantine (65.1%, 30.7%, and 15.3%, for psychological, physical, and sexual violence, respectively) as compared to during the quarantine (50.2%, 13%, 11.2%, respectively) |
| Adibelli,2021 | Turkey | 332 | VAW |  | In the study, some of the women noted that they were exposed to violence by their partners before the pandemic, and the scores of these women in all subscales were found to be higher |
| Aguero,2020 | Peru | 1020 | VAW |  | **Increase**: number of calls increased during 2020, particularly after March |
| Alharbi,2021 | Saudi Arabia | 2254 | VAW | 25.4% before and 16.6% during confinement, indicating an overall decrease of 8.8% in the reported cases | Of the group who experienced multiple forms of violence, 120 (39.9%) reported an **increase** in the frequency and perceived intensity of the violence since the confinement, 128 (42.5%) reported an **unchanged frequency** and intensity of violence. The remaining 53 (17.6%) noted a **reduction** in violent outbursts. |
| Arenas-Arroyo,2020 | Spain | 8951 | VAW |  | We find that during the quarantine, IPV **increased** significantly by 4.5 percentage points (pp, hereafter), equivalent to an increase of 23.38% relative to the pre-lockdown average, which is driven by an increase of the sexual and psychological types of abuses. Instead, we find **no effect** on the level of physical violence. Our findings indicate that both the lockdown and the economic stress cause an independent from each other and significant **increase** in the level of IPV, with the largest effects occurring when both members of the couple are locked together (14–16%) and when both suffer from economic stress (25–33%). |
| Artinopoulou,2021 | Greece | 705 | VAW | Before 19%, during pandemic: 10.7% | **Decrease** |
| Barbara,2020 | Italy |  | VAW | we surprisingly observed a decrease in the number of women who asked in-person assistance and phone counselling at the SVSeD. Specifically, from February 24th to April 21th 2020, the SVSeD offered emergency health care and psychosocial support to 34 women victims of IPV. In the same period last year (2019), we assisted 69 IPV victims. Consistent with the SVSeD experience, the Prosecutor’s office in Milan has confirmed a drastic decrease in criminal proceedings for IPV (personal communication, Deputy Public Prosecutor of Milan). Specifically, from February 21th to April 17th 2020, criminal proceedings for IPV were 178, versus 364 in the same period in 2019. | **Decrease** |
| Barchielli,2021 | Italy | 211 | VAW&M | Specific to DVA between couples: 2019: 85, 2020: 134 reports | **Increase**: statistically significant difference between incidents that occurred in 2019 and 2020 (X2 (1, 341) = 19.240, p = 0.000) |
| Bhattaram,2021 | India | 828 | VAW | 2019: 7 cases (4%), 2020: 34 (7%) | Intimate partner violence also **increased** to 7%. X2 (3, N=662)=21.03, p < .0 |
| Boman,2020 | USA |  | VAW&M |  | To this point, data from the Maumee Police Department demonstrate that there has in fact been an **increase** in substantiated IPV incidents in 2020 (73 incidents; data to May 26, 2020) compared to the same point in the year of 2019 (55 incidents) -->33% **increase** |
| Bourne,2021 | Jamaica | 513 | VAW |  | **Increase**: It indicated being victims of domestic violence (n=160, 31.7%), marginally more reported that these acts have occurred at least frequently during the COVID-19 pandemic (53.1%) compared to before this period (49.0%) |
| Bullinger,2021 | USA |  | VAW&M | The authors compare the identical time periods in 2019 and 2020. 10 weeks prior to the stay-at-home orders (SAH) and 5 weeks during SAH. Importantly, in the weeks leading up to the SAH announcement, there are no statistically significant differences in calls during 2020, compared to 2019, for any outcome. Relative to trends in 2019, estimates indicate that the SAH announcement resulted in a decline in total calls for police service. When looking specifically at most calls for domestic conflicts and crimes, however, we present evidence that calls for domestic violence, domestic disturbance, and domestic battery generally increase in the weeks following the SAH announcement. Calls related to child abuse remain constant. | We find that calls for domestic violence **increased** by 7.4 percent, with larger increases of nearly 9 percent for domestic disturbance calls. These figures correspond to approximately 200 more domestic-related calls across the city of Chicago per week. Notably, we find **no changes** in calls for domestic battery, suggesting that the influx of calls may be related to less severe types of domestic violence, although we cannot rule out increases ranging from 0–12 percent. |
| Campedelli,2020 | USA |  | VAW&M | Results suggest that the policies adopted have not prompted any immediate significant change in intimate partner assaults. The models considering the days from March 4th to March 16th as the period of intervention show **non-significant negative effects** (−4.0% and − 2.5%). The univariate model considering the entire period identifies a small non-significant negative effect (−0.28%). Finally, the multivariate model indicates an **increase in intimate assaults due to the policies (+3.3%); but this increase is not statistically significant.** | For entire policy period: −0.28% for simple model; +3.3% for model with 2 covariates; both **effects are non-significant** |
| Capinha,2021 | Portugal |  | VAW&M |  | **Decrease**: In 2020, the total of occurrences (9483) showed a decrease of 10.99% in comparison with the average of the last 4 years. In comparison with 2019 only, the decrease was 11.29%. |
| Decker,2021 | Kenya | 1217 | VAW&M | Timing of IPV experiences of relative to COVID-19 (n = 67)  Before COVID-19 restrictions only – 29.9  Since COVID-19 restrictions only – 43.3  Both time periods – 26.8 | **Increase** |
| Di Franco,2020 | Italy | 19160 | VAW&M |  | While during the lockdown the percentage of males in the total number of victims of violence **decreased** compared to the non-lockdown period (non-lockdown: 57.14%; lockdown: 43.48%), that of females slightly **increased** (non-lockdown: 41.9%; lockdown: 43.48%). |
| El-Nimr,2021 | Arab women - different countries | 490 | VAW |  | Exposure to any type of IPV during the lockdown has **significantly increased** by 7.3% compared to before the lockdown. The percent of exposure to psychological, physical, and sexual violence has significantly increased during the lockdown compared to before the lockdown, while the increase in the percent of women who reported financial abuse was not significant. Similar proportions of women were exposed to verbal violence before and during the lockdown |
| Erausquin,2021 | Multicountry | 22724 | VAW&M |  | Experiences of intimate partner violence may have **decreased** during COVID-19 measures compared to prior to the pandemic |
| Farmani,2020 | Iran | 20885 | VAW | estimated incident rate of spousal abuse during pandemic of 10.71 per 100000 persons; 520 spousal abuse cases/7319 total calls; 7.1% | These findings showed that the incidence of spousal abuse, child abuse, elder abuse, disability abuse, violence of other relatives, child labor, divorce petition, acute family dispute, unemployment/financial problems, substance abuse, and health questions about COVID-19 coronavirus **increased** significantly during the COVID-19 pandemic |
| Fereidooni,2021 | Iran | 2116 | VAW | 65.4% post-pandemic and 54.2% pre-pandemic - any IPV | 25∙5% (22∙9, 28∙4) of women who didn’t report any experience of IPV before the pandemic reported that they were exposed to at least one episode of IPV during the first six months of the pandemic. The prevalence of IPV **increased significantly** during the first six months of the pandemic. |
| Gibbons,2021 | Argentina | 1502 | VAW |  | **Increase** in calls to helplines: The effect of quarantine on intimate partner violence is generalized. For all three metrics, the point estimates have the expected signs and all coefficients are statistically significant. The size differences are important: focusing on mean effects, we see that emotional violence is 12% higher, sexual violence is 35% higher, and physical violence is 23% higher for women whose partners are also in quarantine. |
| Gleason,2021 | USA | 1051 | VAW&M | Sexual and physical violence was reported by a small number of participants, and therefore statistical analyses were not conducted to determine differences in reported violence before and during the pandemic | Among those who reported being the victim of any sexual or physical violence before or during the pandemic (N = 61), 17 (27.9%) reported that they had experienced **more** physical, sexual, and emotional violence during the COVID-19 pandemic, while 44 (72.1%) **did not report an increase**. Women: experienced more violence during the pandemic, n=8 |
| Gosangi,2020 | USA | 62 | VAW | A total of 62 IPV victims of all types (physical and non-physical abuse) were identified in 2020; 104 in 2019; 106 in 2018; and 146 in 2017 for this seven-week time window. Thus, the overall number of reported IPV victims of all types (including physical and non-physical abuse) during 2020 was 62 victims compared to 342 victims during the prior years (114 per each year), i.e., 0.5 times the incidence in 2020 versus 2017-2019 (95% confidence interval [CI] 0.4 to 0.7, p<0.001). |  |
| Hamadani,2020 | Bangladesh | 2424 | VAW | Emotional violence included insults (reported by 19·9% [95% CI 18·2–21·6]; 290 [68·4%] of 424 reported an increase), humiliation (reported by 8·9% [7·8–10·2]; 126 [66·0%] of 191 reported an increase), and intimidation (reported by 13·5% [12·1–15·0]; 200 [68·7%] of 291 reported an increase). Physical violence (eg, being slapped or having something thrown at them) was reported by 6·5% (5·5–7·6; 76 [56%] of 135 reported an increase). Sexual violence was less common (3·0%, 2·3–3·8), but of those affected, 33 (50·8%) of 65 reported it had increased since the lockdown. | Among women experiencing emotional or moderate physical violence, over half reported it had **increased** since the lockdown |
| Helland,2021 | Norway | 1747 | VAW&M | We found **no indication of increased** interparental conflicts in the lockdown group and rather, the findings suggest somewhat lower verbal aggression during lockdown. We found no effect on physical aggressive conflict behaviors | **Decrease** in verbal agression. |
| Holland,2021 | USA | 187508065 | VAW&M | Counts were significantly lower for IPV ED visits (n = 442 vs 484, P < .001) between March 15 and October 10, 2020, compared with the same period in 2019, | **Weekly ED visit** counts for all 6 outcomes **decreased** between March 8 and 28, 2020 (March 8: MHCs = 42 903, SAs = 5212, all ODs = 14 543, opioid ODs = 4752, IPV = 444, and SCAN = 1090; March 28: MHCs = 17 574, SAs = 4241, all ODs = 12 399, opioid ODs = 4306, IPV = 347, and SCAN = 487); |
| Indu PV,2021 | India | 209 | VAW |  | (Of victims), 11.1 % reported the onset and 5.56 % worsening of DV during the lockdown. |
| Jetelina,2020 | USA | 1730 | VAW&M | The odds of worsening victimization during the pandemic was significantly higher among physical and sexual violence. While the majority of IPV participants reported victimization to remain the same, sexual and physical violence was exacerbated during the early stages of the pandemic. | Among the respondents that screened positive, 54% stated the victimization **remained the same** since the COVID19 outbreak, while 17% stated it **worsened** and 30% stated it **got better**; women, n=25 got worse during covid, n=92 same, n=55 better |
| Krishnamurti,2021 | USA | 959 | VAW | The incidence rates of voluntary IPV screening for new app users during the two time periods were similar (before sheltering in place: 252/552, 46%; during sheltering in place: 163/407, 40%) | The overall use of the IPV screening tool **increased** during the shelter-in-place order. A slight, **nonsignificant increase** in the incidence of physical, sexual, and psychological violence during the shelter-in-place order was found across all app users |
| Lampe,2021 | Austria | 67 | VAW&M | Individuals with prior DV reported significantly higher DV than previously not affected individuals. | However, a statistically significant decrease of DV was found in the group with prior DV. Past DV, childlessness and insecure attachment, but not COVID-19 relate stressors predicted current DV. |
| Lee,2021 | USA | 291 | VAW&M |  | Results indicated that couples’ disagreement and verbal fighting scores **increased** from Time 1 to Time 2, but disagreements related to COVID-19 and **physical fighting did not** |
| Lindau,2021 | USA | 3200 | VAW | 13% during early pandemic; 9% in the 12 months pre-pandemic |  |
| MacCarthy,2020 | USA | 52 | VAW&M |  | 13.5% reported "**increase** in physical conflict or verbal arguments with a partner" with quarantine (not disaggregated by gender) |
| Maftei,2021 | Romania | 1113 | VAW&M |  | in the overall sample, 13.7% of the participants considered that their abusive behaviors **increased** since the pandemic, and the same percentage considered that their partner’s technological abuse also increased since the COVID-19 outbreak – (not disaggregated by gender) |
| Mahmood,2021 | Iraq | 346 | VAW | **Significant increases** in violence were observed from the pre-lockdown period to the lockdown period | Significant **increases** in violence were observed from the pre-lockdown period to the lockdown period for any violence (32.1% to 38.7%, p = .001), emotional abuse (29.5% to 35.0%, p = .005), and physical violence (12.7% to 17.6%, p = .002). Forcing to have sexual intercourse also significantly increased during lockdown (6.6% to 9.5%., p = .021) |
| McMillan,2021 | USA | 49 | VAW |  | 38.3% (18) of expectant mothers reported **increased** verbal arguments and 23.9% (11) reported **increased** physical conflict (self, partner, or both) |
| Muldoon (1),2021 | Canada | 35 | VAW&M | percent (95%CI) difference in Intimate partner assailant: 8.60 (− 11.00, 28.20), p=0.399 |  |
| Ojeahere,2021 | Nigeria | 474 | VAW&M | Statistically significant differences (prior vs during lockdown) were demonstrated for the overall prevalence of IPV (χ2 =14.75, p = .001) and across physical, financial, emotional, and sexual forms of IPV (χ2 =12.66, p = .002; χ2 =7.99, p =.02; χ2 = 22.293, p ≤ .001; χ2 = 9.661, p = .01 | **Decrease**: Using the lockdown as the landmark, higher prevalence was found before than during the lockdown across physical, emotional, financial, and sexual forms of IPV |
| Pal,2021 | India | 271 | VAW&M | CAS-SF scores during lockdown were significantly **higher** than before lockdown | **increase** in IPV during lockdown, compared to before - IPV, more specifically sexual violence, increased in both genders during the lockdown. |
| Pattojoshi,2020 | India | 560 | VAW | 76/560 - experienced similar kind of violence before the lockdown and 25/560 since the lockdown started | If it was there even before, are you experiencing **increased** violence since lockdown? Yes: 59/560; no: 17/560 (Of those who reported spousal violence to be present before lockdown, 77.6% (n = 59) reported an increase in violence since the lockdown was enforced) |
| Peitzmeier,2021 | USA | 1169 | VAW | Prevalence of experiencing any of these forms of IPV in the immediate pre-COVID and during COVID periods did not differ significantly (16.2% vs 15.1%), while 3.3% (33/1145) of the sample reported no incidents of IPV pre-COVID but experienced IPV since COVID, an additional 4.3% (49/1145) reported the reverse—that they were experiencing IPV pre-COVID but had not experienced IPV since COVID—hence the lack of significant net change in the overall prevalence of any IPV since COVID | there were **indications that IPV severity increased** and that novel cases of IPV are occurring in relationships that previously had no abuse |
| Plasilova,2021 | Czech Republic | 429 | VAW | A significant difference was observed in the incidence of IPV in the first wave of the COVID-19 pandemic compared to the pre-pandemic period, as well as in the second wave compared to the pre-pandemic period. A **lower incidence** of IPV was found in both waves of the COVID-19 pandemic compared to the pre-pandemic period. However, in both cases, the effect sizes were small. No significant difference in the incidence of IPV was observed when comparing the first and second waves of the COVID-19 pandemic | **Decrease**: this study suggests that there is a significant difference in the incidence of IPV at 3 months prior to the COVID-19 pandemic compared to the first and second wave of the COVID-19 pandemic |
| Rhodes,2020 | USA | 50 | VAW&M | Domestic violence perpetrators by husbands during the COVID-19 lockdown showed a dramatic reduction during the study periods (33.3% vs. 0.0%) - corresponds to n= 1 vs n=0 | A statistically significant (p = 0.01) increase in assaults was found during the COVID-19 lockdown, particularly during the period after school closures. |
| Sabri,2021 | USA | 45 | VAW | The COVID-19 pandemic was reported to increase frequency and severity of IPV for immigrant women in abusive relationships due to factors such as abusive partners being at home. Other risks reported were increase in gun purchase and decrease in clients seeking legal services | All participants described a reciprocal and reinforcing relationship between increased life stressors and IPV due to the COVID 19 pandemic and associated response |
| Tadesse,2020 | Ethiopia | 589 | VAW | the prevalence of IPV among married women was **comparable to the national pre-COVID figure of IPV** |  |
| Takaku,2021 | Japan | 15836 | VAW |  | we **do not see any significant effect** in other family outcomes, such as incidence of DV or quality of marriage index |
| Teshome,2021 | Ethiopia | 464 | VAW |  | Among the 44 pregnant women who experienced IPV in the year of the interview, 9 (20.4%) reported an increase in IPV after the pregnancy (Table 4) and 8 (18.2%) perceived **increased IPV** after the COVID-19 outbreak |
| Tierolf,2020 | Netherlands | 87 | VAW&M | **No significant difference** was found between families before and during the coronavirus crisis with regard to the percentage of families where ’no’, ’moderate’ or ’serious’ violence occurred |  |
| Vives-Cases,2021 | Spain |  | VAW | Differences between the volume of contacts made via 016-call and the policy reports generated provide evidence for the existence of barriers to IPV-service access during the lockdown and the period of remote working | During Q2 2020, 016**-calls registered an increase** above 45% as compared to Q2 2019 |
| Walsh,2021 | USA | 214 | VAM | 46.88% and 34.44% of IPV victimization and perpetration cases was new or more frequent | **Increase**: Approximately 15%, or 32 individuals, reported victimization during the first 5-8 months of the pandemic (depending on survey response date), of which 50% was new or worsening |

Table S4. Quality Appraisal scores (Joanna Briggs Institute Tools)

| **Author** | **Year** | **Quality appraisal score** | **Quality appraisal category** |
| --- | --- | --- | --- |
| Abrahams et al. | 2022 | 88 | High |
| Abuhammad | 2021 | 50 | Moderate |
| Abujilban et al. | 2020 | 63 | Moderate |
| Adibelli | 2021 | 38 | Low |
| Aguero | 2020 | 50 | Moderate |
| Akalin et al. | 2022 | 38 | Low |
| Akel et al. | 2020 | 88 | High |
| Alharbi et al. | 2021 | 63 | Moderate |
| Arenas-Arroyo et al. | 2021 | 63 | Moderate |
| Artinopoulou | 2021 | 100 | High |
| Barbara et al. | 2020 | 0 | Low |
| Barchielli et al. | 2021 | 100 | High |
| Bhattaram et al. | 2021 | 75 | High |
| Boman et al. | 2020 | 0 | Low |
| Bourne et al. | 2021 | 38 | Low |
| Boxall et al. | 2021 | 63 | Moderate |
| Bueso-Izquierdo et al. | 2021 | 75 | High |
| Bullinger et al. | 2021 | 75 | High |
| Buttell et al. | 2021 | 50 | Moderate |
| Campedelli et al. | 2020 | 88 | High |
| Cannon et al. | 2021 | 38 | Low |
| Cano-Lozano et al. | 2021 | 88 | High |
| Capinha et al. | 2021 | 25 | Low |
| Chen et al. | 2021 | 88 | High |
| Clemens et al. | 2021 | 63 | Moderate |
| Daigle | 2021 | 88 | High |
| Das et al. | 2021 | 63 | Moderate |
| Davis et al. | 2021 | 88 | High |
| Decker et al. | 2021 | 75 | High |
| Dekel et al. | 2021 | 70 | Moderate |
| Di Franco et al. | 2020 | 75 | High |
| Ditekemena et al. | 2021 | 63 | Moderate |
| Ebert et al. | 2021 | 100 | High |
| El-Nimr et al. | 2021 | 75 | High |
| Erausquin et al. | 2021 | 88 | High |
| Farmani et al. | 2021 | 63 | Moderate |
| Fereidooni et al. | 2021 | 100 | High |
| Fleming et al. | 2021 | 100 | High |
| Gama et al. | 2021 | 75 | High |
| Gebrewahd et al. | 2020 | 88 | High |
| Ghimire et al. | 2020 | 25 | Low |
| Gibbons et al. | 2021 | 13 | Low |
| Gillespie et al. | 2021 | 88 | High |
| Gleason et al. | 2021 | 88 | High |
| Gosangi et al. | 2021 | 100 | High |
| Gresham et al. | 2021 | 100 | High |
| Hamadani et al. | 2020 | 75 | High |
| Helland et al. | 2021 | 78 | High |
| Holland et al. | 2021 | 83 | High |
| Indu et al. | 2021 | 88 | High |
| Jetelina et al. | 2020 | 100 | High |
| Johnson | 2021 | 100 | High |
| Karp et al. | 2021 | 100 | High |
| Kimura et al. | 2021 | 80 | High |
| Kliem et al. | 2021 | 50 | Moderate |
| Krishnamurti et al. | 2021 | 50 | Moderate |
| Lampe et al. | 2021 | 100 | High |
| Lee et al. | 2021 | 60 | Moderate |
| Lindau et al. | 2021 | 88 | High |
| Logie et al. | 2021 | 80 | High |
| MacCarthy et al | 2020 | 75 | High |
| Maftei et al. | 2021 | 63 | Moderate |
| Mahmood et al. | 2021 | 75 | High |
| Maxine et al. | 2020 | 88 | High |
| McLean et al. | 2021 | 63 | Moderate |
| McMillan et al. | 2021 | 100 | High |
| Moawad et al. | 2021 | 63 | Moderate |
| Morgan et al. | 2020 | 88 | High |
| Muldoon et al. (1) | 2021 | 75 | High |
| Muldoon et al. (2) | 2021 | 100 | High |
| Naghizadeh et al. | 2021 | 100 | High |
| Nyashanu et al. | 2021 | 80 | High |
| Oginni et al. | 2021 | 100 | High |
| Ogrodniczuk et al. | 2021 | 88 | High |
| Ojeahere et al. | 2021 | 75 | High |
| Olushola et al. | 2021 | 100 | High |
| Overall et al. | 2021 | 89 | High |
| Pal et al. | 2021 | 88 | High |
| Parrott et al. | 2021 | 100 | High |
| Pattojoshi et al. | 2020 | 25 | Low |
| Peitzmeier et al. | 2021 | 100 | High |
| Plášilová et al. | 2021 | 100 | High |
| Radusky et al. | 2021 | 63 | Moderate |
| Rahman et al. | 2020 | 38 | Low |
| Ravi et al. | 2021 | 70 | Moderate |
| Rayhan et al. | 2021 | 100 | High |
| Rhodes et al. | 2020 | 25 | Low |
| Rockowitz et al. | 2021 | 63 | Moderate |
| Rodriguez et al. | 2021 | 80 | High |
| Rojas-Solis et al. | 2021 | 50 | Moderate |
| Sabri et al. | 2020 | 60 | Moderate |
| Schokkenbroek et al. | 2021 | 100 | High |
| Spencer et al. | 2021 | 75 | High |
| Stephenson et al. | 2021 | 100 | High |
| Tadesse et al. | 2021 | 100 | High |
| Takaku et al. | 2021 | 88 | High |
| Tesfaw et al. | 2021 | 50 | Moderate |
| Teshome et al. | 2021 | 100 | High |
| Tierolf et al. | 2021 | 64 | Moderate |
| Vives-Cases et al. | 2021 | 71 | Moderate |
| Walsh et al. | 2021 | 100 | High |
| Wong et al. | 2021 | 100 | High |
| Yari et al. | 2021 | 100 | High |
