# Supplementary material for: Intimate partner violence during COVID-19: systematic review and meta-analysis according to methodological choices
Source: BMC Public Health. 2024 Jan 29;24:313. doi: 10.1186/s12889-024-17802-9 (PMC10823599; doi:10.1186/s12889-024-17802-9)
Supplement: Supplementary file 3 — Additional file 3. Figure S3.1. Forest plot of (“any” type of) intimate partner violence against women (VAW) prevalence pooled by method of administration, between-study variance τ2= 0.00552919. Figure S3.2. Forest plot of (“any” type of) intimate partner violence against men (VAM) prevalence pooled by method of administration (only “online” observed), between-study variance τ2= 0.00729038. Figure S3.3. Forest plot of (“any” type of) intimate partner violence against women & men (VAM) prevalence pooled by method of administration in online and other, between-study variance τ2= .09240205. Figure S3.4. Forest plot of (“any” type of) intimate partner violence against women (VAW) prevalence pooled by type of sample in clinical, convenience or general population/community sample, between-study variance, τ2= 0.00552919. Figure S3.5. Forest plot of (“any” type of) intimate partner violence against men (VAM) prevalence pooled by type of sample (only “convenience” observed), between-study variance, τ2= 0.00729038. Figure S3.6. Forest plot of (“any” type of) intimate partner violence against women and men prevalence pooled by type of sample (only “convenience” observed), between-study variance, τ2= .09240205. Figure S3.7. Forest plot of (“any” type of) intimate partner violence against women prevalence pooled by instrument used for assessment in standardized tool, specifically created questions, between-study variance, τ2= 0.00552919. Figure S3.8. Forest plot of (“any” type of) intimate partner violence against men prevalence pooled by instrument used for assessment in standardized tool and specifically created questions, between-study variance, τ2= 0.00729038. Figure S3.9. Forest plot of (“any” type of) intimate partner violence against women and men prevalence pooled by instrument used for assessment in standardized tool and specifically created questions (from studies where sex-disaggregated prevalence estimates were not available), between-study variance, τ2= 0.09240205. [file 12889_2024_17802_MOESM3_ESM.docx]

**Supplementary material 3 – Only Quality Appraisal = High**

Forest plots with estimates restricted to studies conducted with quality appraisal as high (max n=67)

Figure S3.1 - Forest plot of (“any” type of) intimate partner violence against women (VAW) prevalence pooled by method of administration, between-study variance τ^2^= 0.00552919.

Figure S3.2 - Forest plot of (“any” type of) intimate partner violence against men (VAM) prevalence pooled by method of administration (only “online” observed), between-study variance τ^2^= 0.00729038

Figure S3.3 - Forest plot of (“any” type of) intimate partner violence against women & men (VAM) prevalence pooled by method of administration in online and other, between-study variance τ^2^= .09240205

Figure S3.4 - Forest plot of (“any” type of) intimate partner violence against women (VAW) prevalence pooled by type of sample in clinical, convenience or general population/community sample, between-study variance, τ^2^= 0.00552919

Figure S3.5 - Forest plot of (“any” type of) intimate partner violence against men (VAM) prevalence pooled by type of sample (only “convenience” observed), between-study variance, τ^2^= 0.00729038

Figure S3.6 - Forest plot of (“any” type of) intimate partner violence against women and men prevalence pooled by type of sample (only “convenience” observed), between-study variance, τ2= .09240205

Figure S3.7 - Forest plot of (“any” type of) intimate partner violence against women prevalence pooled by instrument used for assessment in standardized tool, specifically created questions, between-study variance, τ2= 0.00552919

Figure S3.8 - Forest plot of (“any” type of) intimate partner violence against men prevalence pooled by instrument used for assessment in standardized tool and specifically created questions, between-study variance, τ^2^= 0.00729038

Figure S3.9 - Forest plot of (“any” type of) intimate partner violence against women and men prevalence pooled by instrument used for assessment in standardized tool and specifically created questions (from studies where sex-disaggregated prevalence estimates were not available), between-study variance, τ^2^= 0.09240205
